# CD34^+^ Progenitors Promote Ageing‐Associated Valve Sclerosis through the PDGFR–JNK–JUNB Pathway

**DOI:** 10.1002/advs.78607

**Published:** 2026-11-10

**Authors:** Xiaoping Yang, Hong Wu, Guoqiang Ren, Yuesheng Zhang, Zuoshi Wen, Pengwei Zhu, Zhangquan Ying, Liujun Jiang, Tingting Tao, Bohuan Zhang, Linwen Zhu, Han Wang, Han Zhang, Qingbo Xu, Jun Cheng, Honghua Ye

**Affiliations:** ^1^ Department of Cardiology The Affiliated Lihuili Hospital of Ningbo University Ningbo University Ningbo Zhejiang China; ^2^ Department of Geriatrics Xinhua Hospital Shanghai Jiao Tong University School of Medicine Shanghai China; ^3^ Department of Cardiology The First Affiliated Hospital Zhejiang University School of Medicine Hangzhou China; ^4^ Department of Cardiothoracic Surgery The First Affiliated Hospital Zhejiang University School of Medicine Hangzhou China; ^5^ Ningbo Key Laboratory for Innovative Research in Aortic Surgery Diagnosis and Treatment Department of Cardiothoracic Surgery The Affiliated Lihuili Hospital of Ningbo University Ningbo University Ningbo Zhejiang China; ^6^ Department of Vascular Surgery Renji Hospital Shanghai Jiao Tong University School of Medicine Shanghai China; ^7^ Key Laboratory of Medical Electrophysiology of Ministry of Education and Medical Electrophysiological Key Laboratory of Sichuan Province Hemodynamics and Medical Engineering Combination Key Laboratory of Luzhou Southwest Medical University Luzhou China

**Keywords:** aortic valve sclerosis, CD34^+^ cells, myofibrotic remodelling, PDGFR–JNK–JUNB axis

## Abstract

Aortic valve sclerosis (AVS), characterised by fibrocalcific remodelling without significant hemodynamic obstruction, represents a critical window for early clinical intervention; however, its underlying cellular drivers during ageing remain poorly understood. Here, genetic lineage tracing and single‐cell RNA sequencing (scRNA‐seq) techniques were used to examine the temporal and spatial functions of CD34^+^ valvular interstitial cells (VICs) in ageing‐associated AVS. Human scRNA‐seq datasets were used in combination with natural ageing and D‐galactose (D‐gal)‐induced mouse models, and a significant age‐dependent downregulation of CD34 in VICs was identified. Lineage tracing and bone marrow (BM) chimeras confirmed that non‐BM CD34^+^ progenitor VICs (prog‐VICs), rather than bone marrow‐derived cells, serve as the origin of periostin^+^ myofibroblast‐like VICs (myo‐VICs) during ageing. Functionally, the systemic or non‐BM‐specific ablation of CD34^+^ cells or the CD34^+^Periostin^+^ transitional subpopulation markedly attenuated age‐related leaflet thickening and fibrotic remodelling. Mechanistically, the JNK–JUNB signalling pathway, mediated by PDGFR, was found to be crucial for the pathological transdifferentiation of CD34^+^ prog‐VICs into periostin^+^ myo‐VICs. Non‐BM CD34^+^ prog‐VICs were found to aggravate senescence‐associated AVS; thus, targeting the PDGFR–JNK–JUNB axis or preserving the prog‐VIC identity may offer therapeutic avenues to arrest early calcific aortic valve disease (CAVD) progression.

## Graphical abstract

In age‐related aortic valve sclerosis, non‐bone marrow CD34^+^ progenitor valvular interstitial cells (VICs) undergo transdifferentiation into periostin^+^ myofibroblast‐like VICs via the PDGFR‐JNK‐JUNB axis, driving leaflet thickening and fibrosis. Ablating non‐bone marrow CD34^+^ cells attenuates this remodeling. Targeting this axis or preserving progenitor holds promise to halt early calcific aortic valve disease progression.

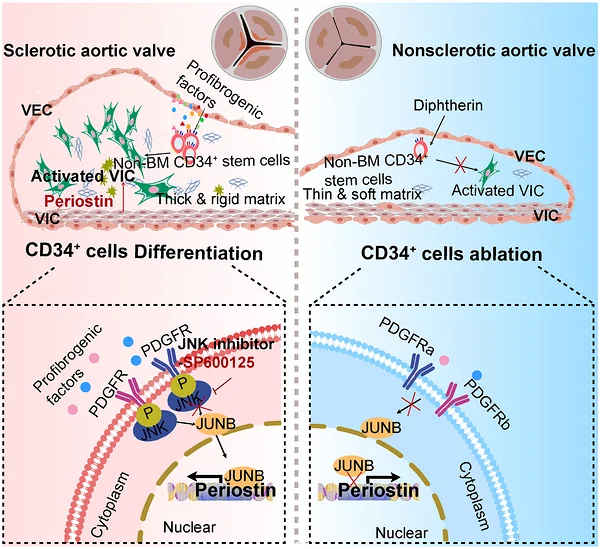

## 1 Introduction

Aortic valve sclerosis (AVS) is the incipient phase of calcific aortic valve disease (CAVD) that involves the fibrocalcific alteration of leaflets without significant impairment of leaflet motion or narrowing of the aortic orifice [1]. Approximately 9% of patients with AVS progress to severe aortic valve stenosis, resulting in significant left ventricular outflow tract obstruction within five years [2]. With the global ageing population, the prevalence of AVS is markedly increasing each year [3]. Other clinical risk factors include male sex, smoking, hypertension, hypercholesterolaemia, obesity, metabolic syndrome, diabetes, and elevated Lp(a) [4]. Notably, the prognosis of obstructive aortic stenosis remains poor unless surgical intervention is performed. Consequently, the identification of therapeutic targets during the “asymptomatic” sclerotic phase is of paramount clinical significance.

Anatomically, the aortic valve (AV) is composed of a specialized three‐layer architecture consisting of the collagenous fibrosa, the glycosaminoglycan‐rich spongiosa, and the elastin‐enriched ventricularis, which are primarily maintained by the valvular interstitial cells (VICs) [5]. Under physiological conditions, VICs exist in a quiescent state that is dedicated to the homeostatic turnover of the extracellular matrix (ECM) [4]. The initiation of AVS is marked by the myofibroblastic differentiation of VICs, a process that follows endothelial injury and inflammatory infiltration [6]. Activated VICs acquire myofibroblast markers, such as *α*‐smooth muscle actin (*α*SMA) and periostin, and secrete abundant amounts of collagen, hyaluronan, and other ECM components into the valvular interstitium [7]. This myofibrogenesis triggers pathological ECM remodelling and leaflet stiffening, which in turn promotes myofibroblastic differentiation [8]. As the disease progresses, cells may eventually undergo osteogenic transition, leading to severe valvular calcification [4, 9]. Multi‐omics analyses by Aikawa et al. highlighted the distinct molecular landscapes occurring between the fibrotic and calcific stages of human AVS, underscoring the significant spatiotemporal heterogeneity and functional plasticity inherent within VICs [10]. Despite the central role of myofibroblast‐like VICs in fibrotic remodelling, their precise cellular origin remains controversial. One prevailing theory suggests that resident VICs are the primary contributors to myofibrogenesis through direct activation [7, 11], while alternative evidence suggests that a specific subset of valvular endothelial cells may undergo endothelial‐to‐mesenchymal transition [12, 13], resulting in a subpopulation of cells with heightened activation propensity. Circulating progenitor cells have also been implicated in valvular disease [14]. Given this complexity, elucidating the specific source of myofibroblast‐like VICs and targeting the molecular drivers of myofibroblast activation during the early fibrotic stage represent a strategic and effective means of halting disease progression before the onset of irreversible calcific remodeling.

Recently, CD34 has emerged as a lineage interface that can identify the tissue‐resident progenitor cells which participate in vascular and cardiac remodeling [15]. Accumulating evidence has demonstrated that CD34^+^ cells function as endothelial and fibroblast progenitor cells (FPCs) and are capable of differentiating into endothelial cells (ECs) [16] and matrix‐producing myofibroblasts [17] under pathological stress. The pivotal role of CD34^+^ FPCs in driving organ fibrosis has been extensively validated across various pathologies, including heart failure [18] and hind limb ischemia [19]. Notably, Lis et al. identified a subpopulation of VICs in normal human aortic valves that co‐express CD34 and PDGFR*α*, the frequency of which is inversely correlated with age and aortic valve degeneration [20]. However, the precise functional significance and lineage fate of these highly heterogeneous CD34^+^ cells during ageing‐associated valvular pathological remodeling remain unexplored. Given the recognized potency of CD34^+^ FPCs in myofibrogenesis, we hypothesized that a CD34^+^ progenitor population within the AV likely serves as a key cellular driver of pathological fibrotic remodeling.

We therefore characterized a distinct fibrotic remodeling phenotype for the AV in both ageing humans and mouse models (natural and D‐gal‐induced ageing) without significant calcific deposition or hemodynamic obstruction and identified a distinct subset of CD34^+^ VICs (progenitor VICs, or prog‐VICs) using scRNA‐seq and multilevel validation. These CD34^+^ prog‐VICs, which comprise approximately 23% of young VICs, undergo profound age‐dependent depletion in terms of both abundance and expression. We then used lineage tracing and a BMT model to demonstrate that non‐bone marrow‐derived CD34^+^ prog‐VICs serve as the source of periostin^+^ myofibroblasts during senescence‐associated fibrotic remodeling. Functionally, the strategic ablation of systemic CD34^+^ cells or non‐BM CD34^+^ cells, and specifically the CD34^+^Periostin^+^ subpopulation, was found to effectively attenuate age‐related leaflet myofibrogenesis, while mechanistically, the PDGFR–JNK–JUNB signaling axis was identified as an indispensable pathway for pathological transdifferentiation. Our study not only resolves the controversy regarding the cellular origins of valvular myofibroblasts but also establishes the PDGFR‐mediated activation of non‐BM CD34^+^ prog‐VICs as a promising therapeutic target that may arrest CAVD at its reversible stage.

## 2 Results

### 2.1 Cellular Senescence‐Induced AV Fibrotic Remodeling

To delineate the morphological evolution of the AV during ageing, human valve samples were collected from individuals spanning all ages from young to elderly and from patients with end‐stage calcification who required surgical intervention (macroscopic images in Figure 1a). Histopathological assessment revealed a progressive increase in the leaflet thickness corresponding to the severity of the CAVD (Figure 1a). Masson's trichrome, PSR, Movat's pentachrome, and Alizarin Red staining demonstrated that, compared with the control samples, sclerotic valves exhibited pronounced collagenous fibrosis and proteoglycan accumulation, whereas mineralized calcium deposition was markedly lower than that observed in the stenotic stage (Figure 1a). These observations were further corroborated in two C57BL/6 murine models which were left to age naturally and the cardiac and valvular alterations compared at 2 months (2‐mo) and 24 months (24‐mo) of age. The results showed that the 24‐mo mice were suffering from significant gross cardiac hypertrophy, as evidenced by increased heart size and heart‐to‐body weight ratios (Figure S1a,b). Histological and semi‐quantitative analyses indicated that aged mice presented with substantial leaflet thickening, ECM remodeling, and proteoglycan enrichment (Figure 1b,c), whereas no significant differences were observed in terms of aortic valvular steatosis or calcification (Figure S1a, middle and bottom). Further, Doppler echocardiographic analysis showed that, despite the absence of significant changes in ejection fraction (EF), transvalvular peak velocity, fractional shortening, or cardiac output, 24‐mo mice exhibited noticeably higher aortic cusp separation (Figure 1d,e) and an increased left ventricular mass (Figure S1d). Furthermore, western blot analysis revealed the upregulation of the senescence marker p53, fibrosis markers (FN1, Collagen I, and Vimentin), and, crucially, markers of VIC activation (periostin and *α*‐SMA) in aged valves (Figure 1f,g and Figure S1l). A second model generated using D‐gal to induce accelerated senescence showed that consistent with our hypothesis, D‐gal‐treated mice developed the hallmark features of AVS, characterized by leaflet thickening, fibrosis, proteoglycan deposition, and elevated expression of senescence‐related and VIC activation‐associated proteins (Figure 1h–m and Figure S1g–k,m). Collectively, these findings suggest that AV fibrotic remodeling occurs naturally during cellular senescence, despite the absence of significant calcific deposition or hemodynamic obstruction.

**FIGURE 1:**
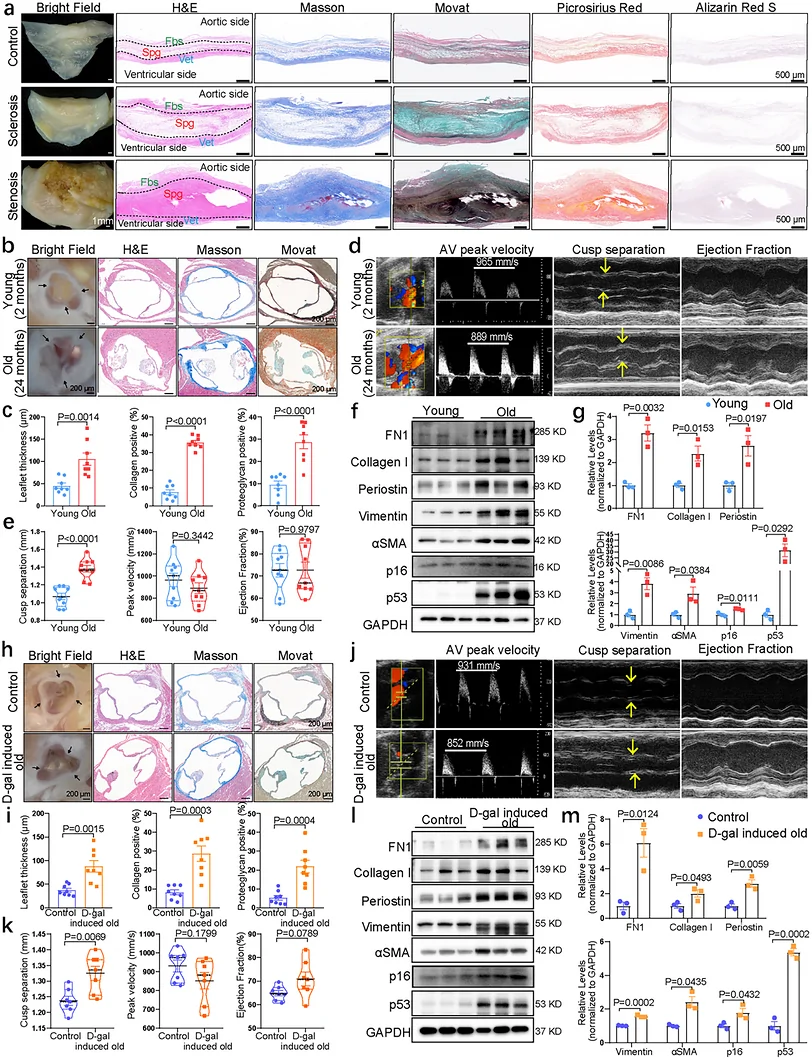
Senescence induces AV fibrosis and ECM deposition. (a) Representative images of the gross pathology and H&E, Masson trichrome (Masson), Movat, picrosirius red (PSR), and Alizarin Red S staining of aortic valves from patients with AVS and stenosis, and the corresponding non‐sclerotic and non‐calcific aortic valve. Scale bars: 1 mm in bright field and 500 µm in pathological staining. (b) Representative images of gross pathology, H&E, Masson and Movat staining of aortic valves from young (2‐mo) and old (24‐mo) mice. Black arrows indicate commissures. Scale bars: 200 µm. (c) Quantification of AV thickness, collagen‐positive fractions, and proteoglycan‐positive fractions in young and old mice (*n* = 8, unpaired two‐tailed Student's t‐test). (d) Representative colour Doppler, spectral Doppler, and M‐mode tracings from young and old mice. (e) Cusp separation, transaortic peak velocity, and EF in young and old mice (*n* = 9, unpaired two‐tailed Student's t‐test). (f) Western blotting of fibrosis‐ and senescence‐related proteins in aortic valves from young and old mice. (g) Quantification of the fold change in indicated proteins from aortic valves in young and old mice (*n* = 3, unpaired two‐tailed Student's t‐test). (h) Representative images showing gross pathology and H&E, Masson, and Movat staining of aortic valves from control and D‐gal‐induced old WT mice. Black arrows indicate commissures. Scale bar: 200 µm. (i) Quantification of AV thickness, collagen‐positive fractions, and proteoglycan‐positive fractions in aortic valves from control and D‐gal‐induced old WT mice (*n* = 8, unpaired two‐tailed Student's t‐test). (j) Representative colour Doppler, Doppler, and M‐mode tracings in the control and D‐gal‐induced old mice. (k) Cusp separation, transaortic peak velocity, and EF in the control and D‐gal‐induced old mice (*n* = 7, unpaired two‐tailed Student's t‐test). (l) Western blotting of fibrosis‐ and senescence‐related proteins in AV from control and D‐gal‐induced old WT mice. (m) Quantification of the fold change in the indicated proteins from the control and D‐gal‐induced aortic valves (*n* = 3, unpaired two‐tailed Student's t‐test). Data are presented as the mean ± SEM. mo: month; WT: wild type; FN1: Fibronectin; D‐gal: D‐galactose.

### 2.2 CD34 Expression Decreased in Ageing‐Associated AVS

Publicly available human single‐cell RNA sequencing (scRNA‐seq) datasets were used to evaluate age‐related changes in CD34 expression of the aortic valve. UMAP‐based dimensionality reduction was found to successfully partition the valvular cells into three major lineages: VICs, ECs, and inflammatory cells (monocytes, macrophages, and T‐lymphocytes) (Figure 2a,b). Subclustering of the VIC population yielded five distinct subsets (Figure 2b). Notably, the proportion of cluster 2 expanded significantly in aged valves compared with the young controls, whereas cluster 3 underwent a marked contraction, with its proportion decreasing from 23.2% to 12.9% (Figure 2c). Based on sub‐cluster‐specific gene expression profiles and functional enrichment analysis, lineage re‐annotation was performed (Figure 2d,e), identifying five distinct VIC subpopulations: homeostatic VICs (Homeo‐VICs), myofibroblast‐like VICs (Myo‐VICs), progenitor VICs (Prog‐VICs), inflammatory VICs (Inflam‐VICs), and osteoblast‐like VICs (Osteo‐VICs). Cluster 3 was characterized by high expression of progenitor‐associated markers such as CD34 (Figure 2d). Pseudotime trajectory analysis further positioned these cells at the root of the differentiation hierarchy, suggesting a primitive, stem‐like state (Figure 2f and Figure S2a–c); hence, this subpopulation was designated prog‐VICs. Conversely, the myo‐VIC cluster was enriched with activated myofibroblast markers, including *ACTA2* and *TAGLN* (Figure 2d). These analyses indicate that prog‐VICs may constitute the predominant population under quiescent conditions, and subsequently undergo transdifferentiation into activated myo‐VICs under pathological stimuli.

**FIGURE 2:**
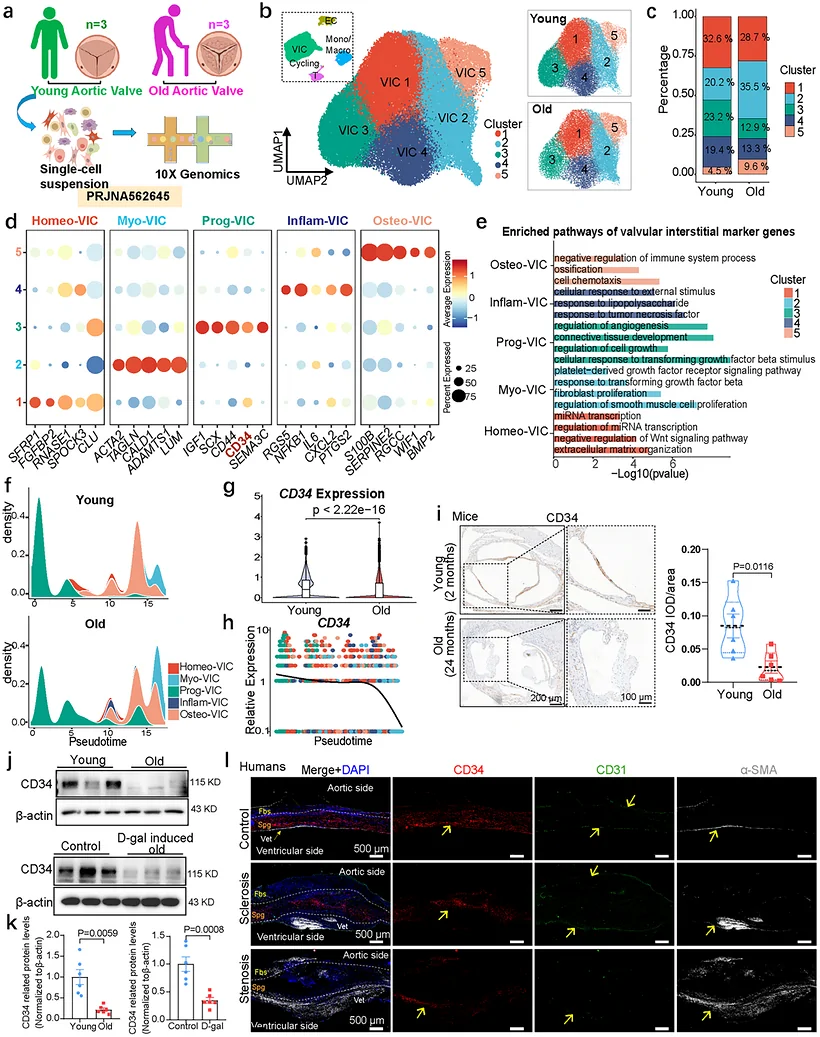
CD34 expression decreases in ageing‐associated AVS. (a) Schematic diagram showing the scRNA‐seq strategy of human aortic valves using data from the public database (PRJNA562645). (b) Left, UMAP plots of integrated scRNA‐seq datasets showing overall cell type and cluster distribution of human aortic valves (top left), and subtype and cluster distribution of valvular interstitial cell subpopulations (bottom left), from young and old patients. Coloured by cluster; Right, split UMAP plots of each group (VICs from young and old groups), coloured by cell cluster. (c) Stack bar plot showing the proportion of VIC subclusters in young and old cells. (d) Dot plot showing the representative DEGs for each VIC subcluster. (e) Bar plot showing the main enriched biological process in each VIC subcluster based on the significant DEGs from each subcluster. (f) Density plot of VIC subclusters in young and old groups across the pseudotime trajectory, coloured by VIC subcluster. (g) Violin plots displaying *CD34* gene expression levels in the VIC subcluster for young and old groups. Statistical *p* values from Wilcoxon testing are added between the two groups. (h) *CD34* gene expression trends across the pseudotime trajectory. (i) Representative immunohistochemical (IHC) images illustrating CD34 expression pattern in young and old aortic valves from mice, with magnified views of boxed regions. Dot plot (right) displayed the average optical density of CD34‐positive staining in aortic valves across different groups (*n* = 6, unpaired two‐tailed Student's t‐test). Scale bars: 200 µm and 100 µm. (j) Western blotting of CD34 expression in aortic valves from young and old mice (upper panel), and control and D‐gal‐induced old mice (lower panel). (k) Quantification of fold changes associated with CD34 expression in AV tissues from young and old mice (left), and control and D‐gal induced old mice (right) (*n* = 3, unpaired two‐tailed Student's t‐test). (l) Immunofluorescence (IF) staining demonstrating CD34 (red), CD31(green), and *α*SMA (grey) expression in AV tissues from human with sclerosis and stenosis, together with non‐sclerotic and non‐calcific AV tissues. Scale bars: 500 µm. Data are presented as the mean ± SEM. scRNA‐seq, single‐cell RNA sequencing; VICs, valvular interstitial cells; DEGs, differentially expressed genes.

Intriguingly, CD34 expression levels were significantly lower in the aged group than their young counterparts (Figure 2g). Pseudotime modelling revealed the progressive downregulation of CD34 during the transition from prog‐VICs to myo‐VICs (Figure 2h and Figure S2d). To corroborate these bioinformatic findings, IHC and western blot analyses were performed on the murine models, with results showing consistent reduction in the protein levels of CD34 in both naturally aged and D‐gal‐induced sclerotic valves (Figure 2i–k and Figure S2e). This trend was further validated in human specimens, which indicated a steady age‐dependent decline in CD34 expression that was inversely correlated with the valvular remodelling progression (Figure 2l and Figure S2f). Our findings demonstrate that CD34 expression in the AV is significantly downregulated in age‐related sclerosis, suggesting that CD34^+^ prog‐VICs may function as a critical population of fibroblast progenitors that participate in the fibrotic remodelling of the aortic valve.

### 2.3 Lineage Tracing Revealed the Contribution of CD34^+^ Prog‐VICs into Periostin^+^ Myo‐VICs

A *Cd34*‐CreER;tdTomato lineage‐tracing mouse model was used to further elucidate the role of CD34^+^ cells in the age‐associated AV remodeling (Figure S3a), for which tamoxifen induction was initiated in 2‐mo adult mice (Figure 3a). As expected, the CD34‐derived (tdTomato^+)^ cells constituted a heterogeneous population under homeostatic conditions, with valve CD34^+^ cells primarily composed of CD31^+^ ECs and plenty of PDGFRa^+^ VICs and a minimal CD45^+^ inflammatory component (Figure S3b–d). While flow cytometry revealed a significant reduction in the number of circulating CD34‐lineage cells in the peripheral blood of the 24‐mo mice (Figure 3b), whole‐mount imaging of the aortic valves demonstrated a marked accumulation of CD34‐orginated cells but a reduced number of CD31^+^ ECs (Figure 3c). Immunofluorescence (IF) staining further validated that myofibroblast markers such as periostin were virtually undetectable in the young aortic valves, and no colocalization was observed between tdTomato (tdT) and periostin (Figure 3d). In contrast, aged aortic valves exhibited a robust accumulation of tdT^+^ cells (CD34‐lineage derived) within the thickened fibrotic interstitium (Figure 3d,e). These lineage‐traced cells demonstrated significant and increasing co‐localization with periostin, indicating their active transdifferentiation into a myofibroblastic phenotype. Quantitative analysis revealed that the tdT^+^/periostin^+^ dual‐positive cells accounted for approximately 51% of the total periostin^+^ myo‐VIC population in the aged valves (Figure 3f). These findings provide definitive in vivo evidence that VICs express minimal levels of periostin under physiological conditions and that nearly half of the pathological periostin^+^ myo‐VICs driving age‐associated valvular fibrogenesis originate from the CD34^+^ progenitor pool.

**FIGURE 3:**
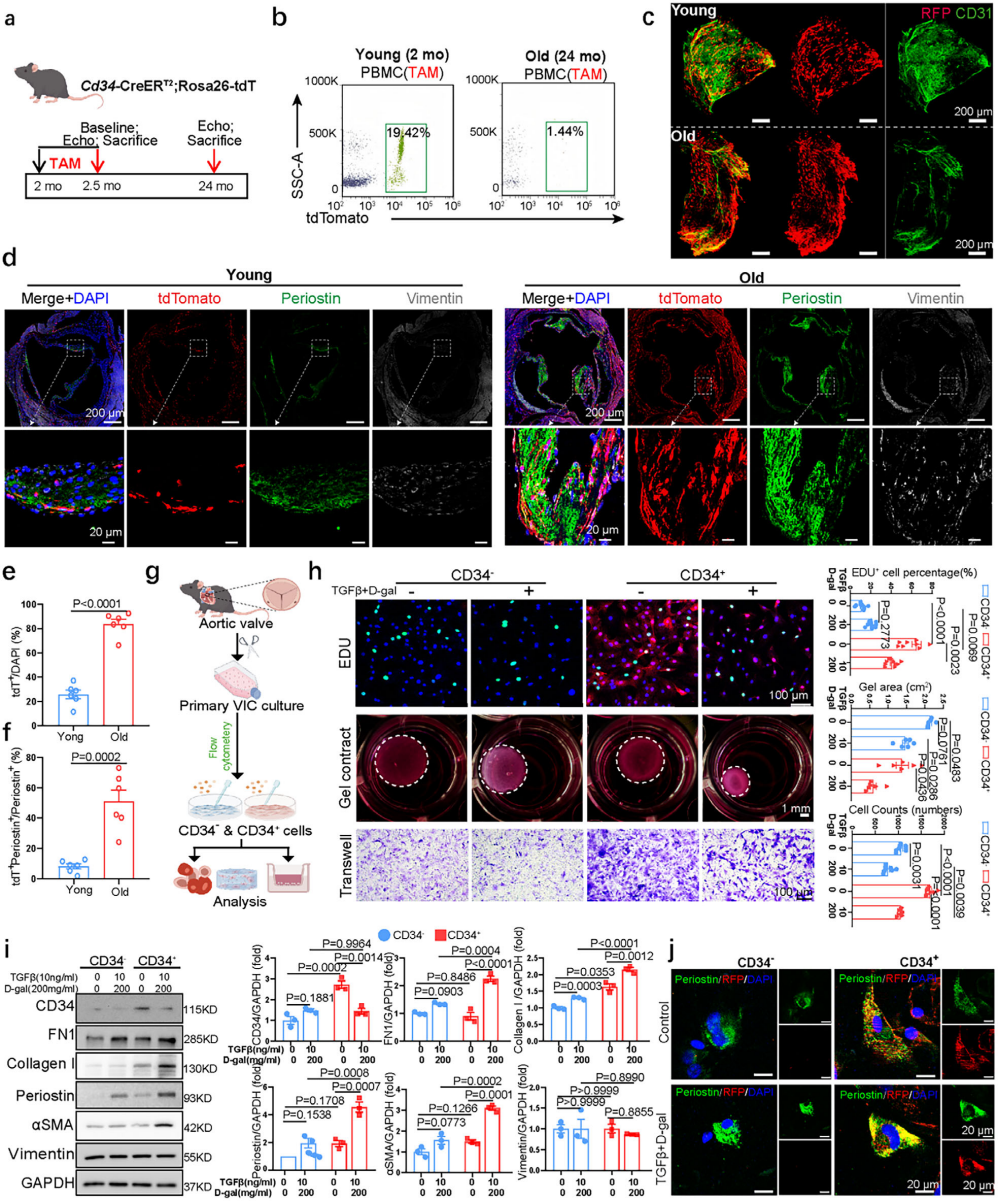
Lineage tracing revealing the contribution of CD34^+^ cells to myofibroblasts. (a) Schematic showing genetic lineage tracing using *Cd34*‐CreER^T2^;R26‐tdT mice. (b) Flow cytometric quantification comparing the percentage of tdTomato (tdT)^+^ cells in PBMCs isolated from young (2‐mo) and old (24‐mo) *Cd34*‐CreER^T2^; R26‐tdT mice. (c) Representative whole‐mount immunostaining images showing tdTomato and CD31 in AV leaflets from young (2‐mo) and old (24‐mo) *Cd34*‐CreER^T2^; R26‐tdT mice. Scale bar, 200 µm. (d) Representative images of tdTomato, co‐staining with the activation of fibroblast marker periostin, and mesenchymal cell marker vimentin in young (2‐mo) and old (24‐mo) *Cd34*‐CreER^T2^; R26‐tdT mice, with boxed region showing magnification. Scale bars: 200, and 20 µm in magnification images. (e) Quantification of remaining tdT^+^ cells in AV sections, illustrating the fraction of tdT^+^ cells that participated in AV remodelling in the indicated group (*n* = 6, unpaired two‐tailed Student's t‐test). (f) Quantification of the percentages of Periostin^+^tdT^+^ cells in the Periostin^+^ cells in the indicated group (*n* = 6, unpaired two‐tailed Student's t‐test). (g) Schematic representation of primary CD34^+^ VICs isolation and the experimental strategy. (h) EDU assay showing cellular proliferation in aortic valvular CD34^−^ and CD34^+^ cells isolated from *Cd34*‐CreER^T2^;R26‐tdT mice, followed by TGF‐*β* (10 ng/mL) and D‐gal (200 µg/mL) stimulation, scale bar: 100 µm. Dotplot (right) displaying the percentages of EDU^+^ cells in primary valvular cells across different groups (*n* = 10, two‐way ANOVA followed by Tukey post hoc test); Representative photographs of collagen gels taken at 18 h showing cells after TGF‐*β* (10 ng/mL) and D‐gal (200 µg/mL). Scale bar: 1 mm. Quantification of gel diameters in indicated groups (right) (*n* = 6, two‐way ANOVA followed by Tukey post hoc test); Representative transwell image showing cellular migration capacity of indicated cells in response to TGF‐*β* (10 ng/mL) and D‐gal (200 µg/mL). Scale bar: 100 µm. Quantification (right) of cell counts in indicated groups (*n* = 6, two‐way ANOVA followed by Tukey post hoc test). (i) Western blot showing the expression of CD34 and fibrosis‐related proteins in the indicated groups, with GAPDH as a loading control. Quantifications are shown on the right (*n* = 3, two‐way ANOVA followed by Tukey post hoc test). (j) Representative IF staining images for periostin in indicated cells following TGF‐*β* (10 ng/mL) and D‐gal (200 µg/mL) stimulation for 12 h. Scale bar: 20 µm. Data are presented as the mean ± SEM. R26, Rosa26; PBMC, peripheral blood mononuclear cells; CD31: Cluster of differentiation 31.

To distinguish between the contributions of BM‐and non‐BM‐derived CD34^+^ cells, BM chimera (BMT) models were established (Figure S4a), with successful reconstruction confirmed using flow cytometric analysis (Figure S4b). Approximately 49.6% of the periostin^+^ myofibroblasts and 15.3% of CD31^+^ ECs in the *Cd34*‐CreER^T2^;Rosa26‐tdT (Donor: C57BL/6) chimeras (tracing non‐BM cells) were tdTomato^+^ (Figure S4c,g,i), with no colocalization observed for CD45 (Figure S4e,i). Conversely, 47.3% of the inflammatory cells in the C57BL/6 (Donor: *Cd34*‐CreER^T2^;Rosa26‐tdT) chimeras (tracing BM‐derived cells) originated from the CD34 lineage (Figure S4f,i), and no periostin^+^ or CD31^+^ cells were tdTomato^+^ (Figure S4d,h,i). These results collectively demonstrate that CD34^+^ cells of BM origin differentiate primarily into inflammatory lineages, whereas non‐BM CD34^+^ prog‐VICs serve as a source of periostin ^+^ myo‐VICs (Figure S4j).

To recapitulate the pathological process of senescence and fibrosis, human immortalized HVICs were treated with a combination of TGF‐*β* and D‐gal (Figure S5a). IF staining confirmed the expression of vimentin and *α*SMA in HVICs, while the endothelial marker CD31 remained absent (Figure S5b), and bulk RNA sequencing identified the significant enrichment of senescence‐ and myofibrosis‐associated gene signatures, validating the successful establishment of the model (Figure S5c). Consistent with our in vivo findings, pathological induction led to the downregulation of CD34 and diminished proliferative capacity (Figure S5c). To refine the rational basis of this dual‐stimulation regimen, we systematically dissected the individual versus combined effects of D‐gal (senescence stress) and TGF‐β (profibrotic stimulus). SA‐β‐gal staining demonstrated that both D‐galactose alone and TGF‐β alone significantly increased the proportion of senescent cells, indicating that TGF‐β itself also promotes cellular senescence in CD34^+^ VICs. Strikingly, co‐treatment synergistically amplified SA‐β‐gal activity (Figure S5f). Moreover, Western blot analysis revealed that Periostin showed a modest upward trend without statistical significance under TGF‐β alone and a slight increase under D‐gal, whereas combined stimulation markedly synergized to drive a robust upregulation of its expression (Figure S5g). Furthermore, protein‐level evaluation confirmed that CD34^+^ VICs underwent marked myofibrogenic and senescent transition upon TGF‐β/D‐gal stimulation, accompanied by the concurrent loss of CD34 and proliferative markers (Figure S5h). To further dissect the functional heterogeneity, the CD34^+^ and CD34^−^ subpopulations were FACS‐sorted from the primary mouse VICs (Figure 3g and Figure S5e). Functional assays revealed superior contractile capacity and increased proliferative and migratory potential for the CD34^+^ VICs as compared to their CD34^−^ counterparts under pathological stimuli (Figure 3h). Western blot and semi‐quantitative analysis revealed significantly higher levels of canonical myofibroblastic markers in CD34^+^ cells, with *α*SMA, periostin, and key fibroblastic structural proteins such as FN1 (Fibronectin 1), collagen I, and vimentin observed relative to CD34^−^ VICs (Figure 3i). IF staining of periostin further highlighted its enhanced myofibrogenic potential (Figure 3j).

### 2.4 Deletion of CD34^+^ Cells Improves Age‐Related Aortic Valvular Fibrotic Remodeling

To functionally determine the role of CD34^+^ cells in the progression of AV fibro‐remodeling, *Cd34*‐CreERT2;R26‐tdT/DTR mice (Figure S6a) were subjected to the experimental procedures depicted in Figure 4a to selectively ablate the CD34^+^ population during D‐gal‐induced AVS. Echocardiographic analysis revealed no significant differences in cardiac function, indicated by EF, peak velocity, and aortic cusp separation, between control and d‐gal‐treated aged mice following systemic CD34^+^ cell ablation (Figure 4b–e and Figure S6b–f), suggesting that functional impairment may have lagged behind the structural remodeling. However, histopathological assessment demonstrated that the ablation of CD34^+^ cells significantly attenuated leaflet thickening, fibrosis, and proteoglycan deposition (Figure 4f). Notably, a significant presence of tdT^+^ periostin^+^ myo‐VICs was observed in the thickened aortic valves of the CD34^+^ non‐deletion group (Figure 4g). In contrast, the aortic valves in the CD34^+^‐deletion group had fewer tdT^+^ cells and fewer tdT^+^ periostin^+^ myo‐VICs (Figure 4g). Moreover, western blot analysis of AV tissues confirmed that the ablation of CD34^+^ cells led to a significant decrease in the expression of myofibroblast‐associated proteins (*α*SMA and periostin) and a marked downregulation of fibrosis‐related markers such as collagen I and vimentin (Figure 4h and Figure S6g). These molecular changes are consistent with the observed attenuation of histological remodeling in CD34‐depleted valves.

**FIGURE 4:**
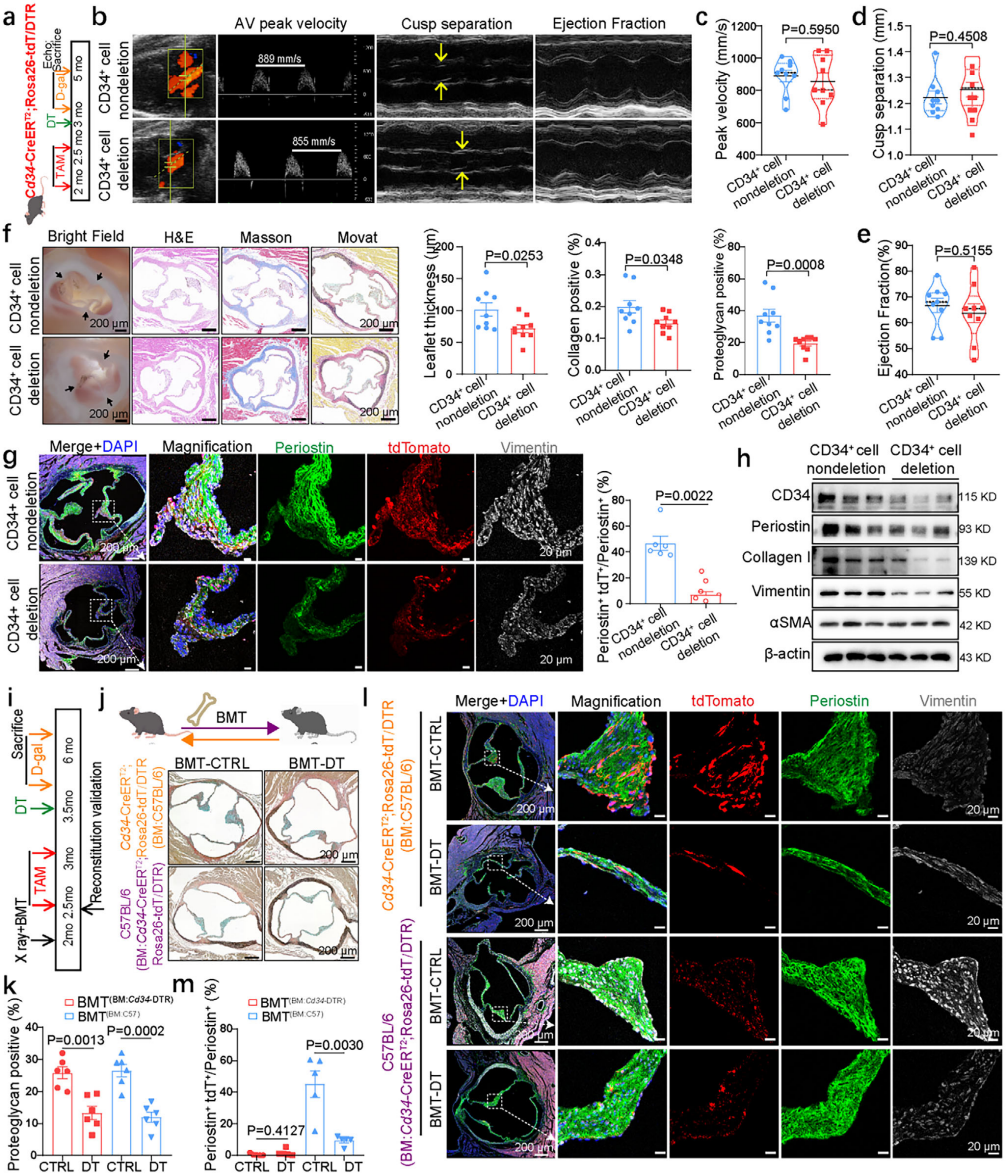
Deletion of CD34^+^ cells decreases the thickness and fibrosis of the ageing‐ related aortic valve. (a) Schematic diagram showing the experimental design for systemic CD34^+^ depletion in *Cd34*‐CreER^T2^;R26‐tdTomato/DTR mice. (b) Representative colour Doppler, spectral Doppler, and M‐mode tracings of CD34^+^ cell deletion group and non‐deletion group following D‐gal injection over 2 months. (c–e) Cusp separation, transaortic peak velocity, and EF in the indicated groups (n = 9, unpaired two‐tailed Student's t‐test). (f) Representative images showing gross pathology and H&E, Masson, and Movat staining of aortic valves from CD34^+^ cell deletion and non‐deletion groups. Black arrows indicate commissures. Scale bar: 200 µm. Quantification (right) of AV thickness, collagen positive fractions and proteoglycan positive fractions in aortic valves from the indicated groups (*n* = 9, unpaired two‐tailed Student's t‐test). (g) IF staining of periostin, tdTomato, and vimentin in AV sections from CD34^+^ cell deletion and non‐deletion groups after d‐gal induction over 2 months, with magnified boxed regions showing split channels to the right. Scale bars: 200 µm and 20 µm. Quantification of the percentage of periostin^+^ tdT^+^ cells among periostin^+^ cells (*n* = 6, unpaired two‐tailed Student's t‐test). (h) Western Blot analyses of CD34 and fibrosis‐related proteins in the indicated groups; Quantifications shown in Figure S6g. (i) Schematic diagram showing the experimental design for non‐BM and BM CD34^+^ cell deletion in indicated chimeric mice using BM transplantation (BMT), DT injection, Tamoxifen, and D‐gal induction, respectively. (j) Representative images obtained using Movat staining of aortic valves from indicated groups. Scale bar: 200 µm. (k) Quantification of AV proteoglycan‐positive fractions in aortic valves from the indicated groups (*n* = 6, unpaired two‐tailed Student's t‐test). (l) IF staining for periostin, tdTomato, and vimentin in AV sections from the indicated groups following D‐gal induction for 2 months, with magnified boxed regions showing split channels to the right. Scale bars: 200 µm and 20 µm. (m) Quantification of the percentage of Periostin^+^tdT^+^ cells in Periostin^+^ cells (*n* = 5, unpaired two‐tailed Student's t‐test). Data are presented as mean ± SEM.

BMTs were then employed in a D‐gal‐induced AVS model to distinguish the specific contributions of CD34^+^ cells based on their origin. The targeted depletion of non‐BM CD34^+^ cells effectively mitigated the AV thickening and diminished the accumulation of proteoglycans and fibrous matrix (Figure 4i–k and Figure S7a,b). Consistent with these findings, a significant reduction in the CD34‐lineage‐derived periostin^+^ myo‐VICs was observed (Figure 4l,m). In contrast, ablation of the BM‐derived CD34^+^ cells yielded no significant changes in the leaflet morphology or fibrotic markers as compared to the control, with no BM‐derived cells (tdTomato^+^) expressing periostin and PDGFRa (Figure 4j–m and Figure S7a–c). Collectively, these results demonstrate that non‐BM CD34^+^ cells, rather than their BM‐derived counterparts, function as prog‐VICs, and that the non‐BM‐derived progenitors participate in the pathological fibrotic remodelling of AV leaflets by transdifferentiating into periostin^+^ myo‐VICs during the ageing process.

Next, an intersectional genetic strategy employing dual recombinase‐based lineage tracing [21] was used to fate map the subpopulation of CD34^+^ cells (CD34^+^Periostin^+^ cells) in senescence‐related AVS and investigate their potential contribution to valve remodeling. To generate *Cd34*‐Dre; *Postn*‐CreER^T2^;Dou‐tdT‐DTR mice for double‐lineage tracing and depletion, *Cd34*‐Dre and *Postn*‐CreER^T2^ mice were crossbred with R26‐RSR‐LSL‐tdTomato‐DTR reporter (R26‐rox‐stop‐rox‐loxp‐stop‐loxp‐tdTomato‐DTR) mice (Figure 5a). The resulting *Cd34*‐Dre;*Postn*‐CreER^T2^;Dou‐tdT‐DTR mice were concurrently administered tamoxifen and DT from 2 until 24 months of age, with the simultaneous application of the Cre‐LoxP/Dre‐Rox recombination systems allowing the specific targeting and ablation of the emerging CD34^+^Periostin^+^ myofibroblast subpopulation during the onset of valvular remodeling (Figure 5b). The results indicated that the deletion of the CD34^+^Periostin^+^ myo‐VICs effectively mitigated the fibro‐remodeling of the aortic valve, as evidenced by the reduced thickness of the valvular leaflets, decreased ECM accumulation, and proteoglycan deposition (Figure 5c). Echocardiographic assessment revealed no significant differences in the cardiac and valvular functions of the DT‐treated and non‐DT groups (Figure 5d–g and Figure S8a–e). However, compared with the DT group, many more tdT^+^ cells co‐expressing periostin were detected in the valvular region of the no‐DT group. Semi‐quantitative analysis revealed that CD34^+^Periostin^+^ myo‐VICs accounted for 22.76% ± 2.99% of the Periostin^+^ myo‐VICs (Figure 5h), and the results therefore indicate that the CD34^+^ prog‐VICs that could be activated and transdifferentiated into periostin^+^ myo‐VICs, promote the progression of ageing‐related aortic valvular fibro‐remodeling.

**FIGURE 5:**
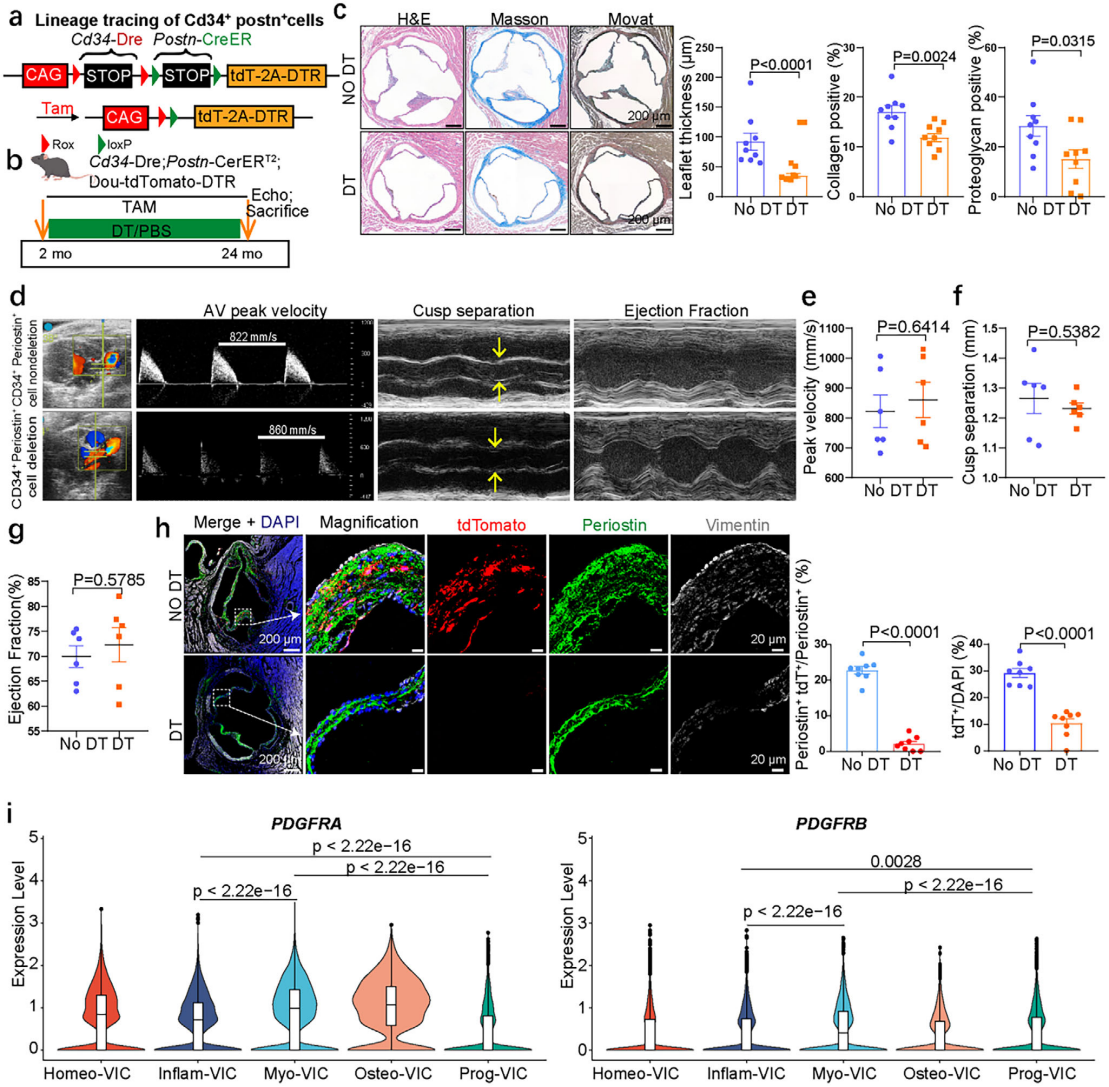
CD34^+^ cells generated Periostin^+^ valve interstitial cells involved AV remodelling. (a) Schematic diagram illustrating the construction strategy of *Cd34*‐Dre;*Postn*‐Cre ER^T2^;Dou‐tdT‐DTR mice. (b) Schematic diagram showing the experimental design for genetic tracing and selective deletion of CD34^+^ cell‐derived Periostin^+^ cells using dual recombinase‐ based lineage tracing in *Cd34*‐Dre;*Postn*‐CreERT2; Dou‐tdT‐DTR mice. (c) Representative images of H&E, Masson, and Movat staining of aortic valves from CD34^+^Periostin^+^ cell deletion group (DT) and non‐deletion group (No DT). Quantification (right) of AV thickness, collagen‐positive fractions and proteoglycan‐positive fractions in the indicated group (*n* = 8, unpaired two‐tailed Student's t‐test). Scale bars: 200 µm. (d) Representative colour Doppler, spectral Doppler, and M‐mode from tracings of *Cd34*‐Dre;*Postn*‐CreER^T2^; Dou‐tdT‐DTR mice with or without DT injection. (e–g) Cusp separation, transaortic peak velocity, and EF in the indicated group (*n* = 6, unpaired two‐tailed Student's t‐test). (h) IF staining for tdTomato, periostin, and vimentin in the AV from CD34^+^Periostin^+^ cell deletion (DT) and non‐deletion (No DT) groups. The boxed regions are magnified with the split channels. Quantification (right) of tdToamto (tdT)^+^ cells in periostin^+^ cells and the remaining tdT^+^ cells in AV sections, illustrating the efficiency of CD34^+^ cell deletion in the indicated groups (*n* = 8, unpaired two‐tailed Student's t‐test). Scale bars: 200 and 20 µm. (i) Violin plots displaying the mRNA expression levels of *PDGFRA* (left) and *PDGFRB* (right) across subclusters. Statistical comparisons were performed specifically among Prog‐VIC, Myo‐VIC, and Osteo‐VIC subclusters. P‐values for these specific comparisons are indicated above the brackets by Wilcoxon test. Data are presented as the mean ± SEM.

### 2.5 Activation of CD34^+^ Prog‐VICs via PDGFR Signaling

To explore the molecular cues driving the activation of CD34^+^ prog‐VICs, we focused on the PDGFR signaling pathway, a well‐recognised mediator of organ fibrosis [22]. Analysis of human AV scRNA‐seq data revealed a distinct expression pattern in which myo‐VICs exhibited markedly elevated levels of both *PDGFRA* and *PDGFRB* compared to the prog‐VIC subpopulation (Figure 5i). This differential expression profile suggests that the induction of PDGFR signaling may serve as a critical trigger for the pathological transition from a progenitor state to a myofibroblast phenotype during valvular remodeling. To further validate the function of PDGFRs during age‐related AVS progression, *Cd34*‐CreER^T2^;R26‐tdTomato mice were crossed with *Pdgfra* ^fl/fl^ or *Pdgfrb* ^fl/fl^ mice to generate *Cd34*‐CreER^T2^; *Pdgfr* ^fl/fl^;R26‐tdTomato mice with knocked out PDGFRa or PDGFRb, specifically in the CD34^+^ cells (Figure 6a,b and Figure S9a–c)). Histopathological assessment and semi‐quantitative statistical analysis revealed that the genetic deletion of both *Pdgfra* and *Pdgfrb* significantly attenuated age‐related valvular remodeling, which manifested as reduced leaflet thickness and diminished ECM and proteoglycan deposition (Figure 6c). Despite the significant attenuation of valvular remodeling, the CD34^+^ cell‐specific *Pdgfra* or *Pdgfrb* knockout did not result in any observable alterations in the global cardiac and valvular function. Quantitative measurements of the EF and fractional shortening in both *Pdgfra^CD34–KO^* and *Pdgfrb^CD34–KO^* aged mice remained comparable to those in young control cohorts (Figure S10a–d). Furthermore, Doppler flow analysis showed no significant differences in the peak aortic jet velocity, valve pressure gradients, or heart/body weight of the groups (Figure S10e–i), suggesting that while PDGFR genetic deletion effectively halts the pathological thickening of the leaflets, the intervention does not adversely affect the baseline cardiac hemodynamics in the aged mouse model. Notably, immunofluorescence staining showed a marked reduction in the co‐localization of tdTomato and periostin in *Pdgfr^CD34–KO^* mice (Figure 6d), providing in vivo evidence that PDGFR*α* and PDGFR*β* are essential for the transdifferentiation of CD34^+^ prog‐VICs within the ageing valvular microenvironment. These findings were further validated in vitro using primary CD34^+^ VICs transfected with siRNAs to achieve the targeted knockdown of *Pdgfra* and *Pdgfrb*. EdU incorporation and transwell assays demonstrated that the downregulation of either receptor via siRNA‐mediated knockdown significantly inhibited CD34^+^ cell proliferation and migration upon TGF*β*/D‐gal stimulation (Figure 6f,g). Importantly, the simultaneous knockdown of both receptors elicited a synergistic inhibitory effect (Figure 6f,g). Thus, to examine the impact on mechanical functionality, a collagen gel contraction assay was performed to assess the effect of PDGFRs on the ECM remodeling capability of these cells. The results showed that the siRNA‐mediated knockdown of *Pdgfra* or *Pdgfrb* significantly reduced the contractile activity induced by TGF‐*β*/D‐gal (Figure 6f,g). Furthermore, molecular characterization revealed that PDGFRs knockdown suppressed the expression of periostin as well as other fibrosis‐related markers, including collagen I, FN1, and vmentin (Figure 6h,i and Figure S10j–l). Collectively, these results indicate that PDGFR is indispensable for the myofibrogenic transdifferentiation of CD34^+^ prog‐VICs during age‐related AV remodelling.

**FIGURE 6:**
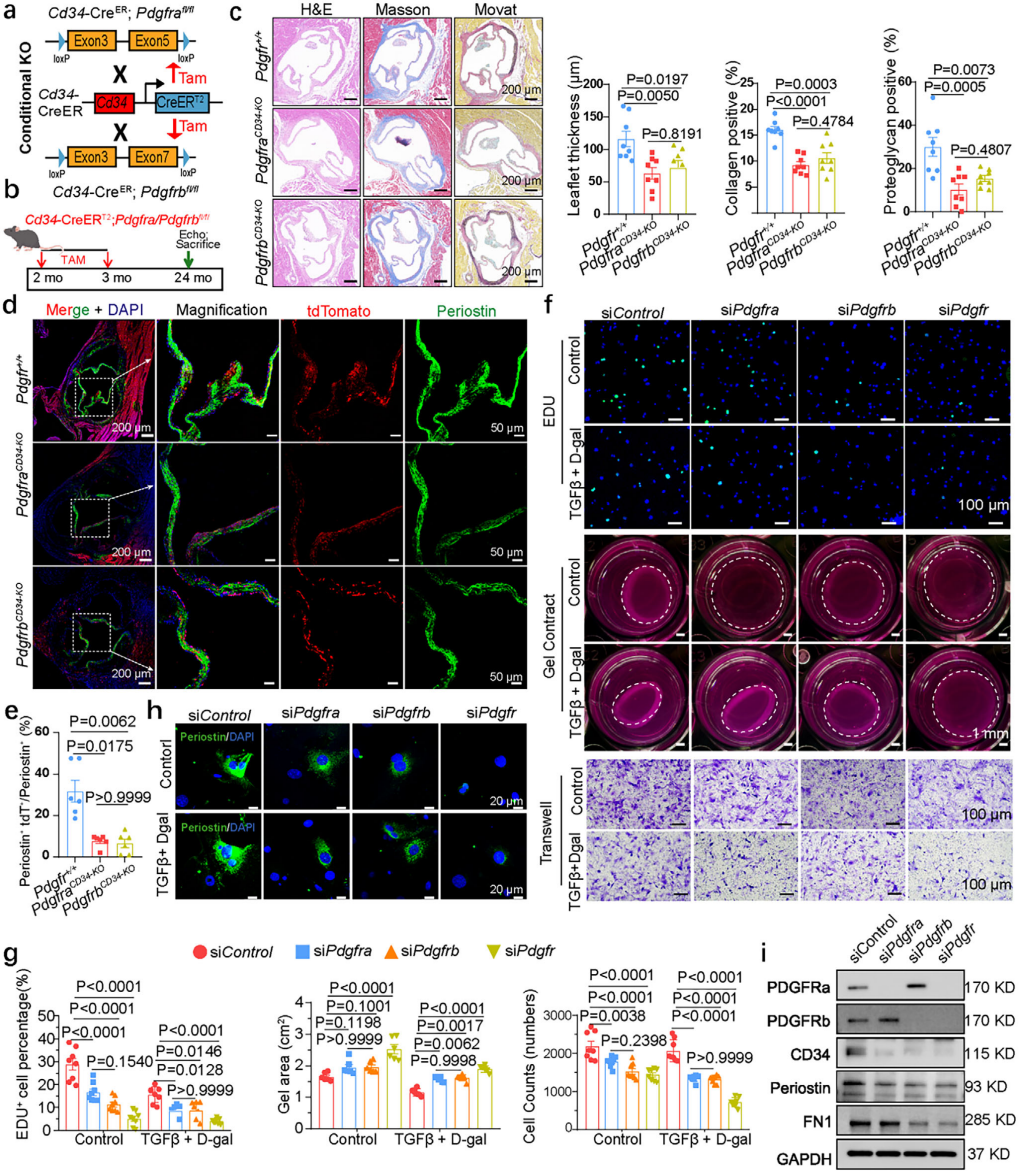
Specific deletion of PDGFRa or PDGFRb in CD34^+^ cells significantly decreases AV remodelling. (a) Schematic image showing the generation of CD34^+^ cell specific *Pdgfra* (*Pdgfra* ^−/−^) or *Pdgfrb* (*Pdgfrb^−/−^*) knockout lineage tracing mouse strain. (b) Schematic diagram showing the experimental design for genetic tracing and specific *Pdgfra* (*Pdgfra^−/−^*) or *Pdgfrb* (*Pdgfrb^−/−^*) knockout in CD34+ cells. (c) Representative images for H&E, Masson, and Movat staining of aortic valvular sections from *Pdgfra* ^CD34–KO^, *Pdgfrb* ^CD34–KO^ and *Pdgfr^+/+^* (*Cd34*‐CreER^T2^;R26‐tdT) mice. Scale bar: 200 µm. Quantification (right) of AV thickness, collagen‐positive fractions, and proteoglycan‐positive fractions in aortic valves from the indicated groups (*n* = 8, two‐way ANOVA followed by Tukey post hoc test). (d) IF staining of periostin and tdTomato in aortic valvular sections from *Pdgfra* ^CD34–KO^, *Pdgfrb* ^CD34–KO^ and *Pdgfr^+/+^* mice. Boxed regions are magnified with split channels. Scale bars: 200 and 50 µm. (e) Quantification of the percentage of tdT^+^Periostin^+^ cells in Periostin^+^ cells for indicated group (*n* = 8, two‐way ANOVA followed by Tukey post hoc test). (f) EDU assay, collagen gel contraction assay, and transwell assay showing cell proliferation, migration, and contraction ability respectively. Primary CD34^+^ VICs cells response to *siPdgfra* or/and *siPdgfrb* treatment, with or without TGF‐*β* (10 ng/mL) and D‐gal (200 µg/mL) stimulation. Scale bar: 100 µm and 1 mm. (g) Dotplot (left) displaying the percentages of EDU^+^ cell in primary valvular cells across different groups (*n* = 8, two‐way ANOVA followed by Tukey post hoc test); Quantification (medium) of gel diameters in indicated groups (*n* = 5, two‐way ANOVA followed by Tukey post hoc test); Quantification (right) of cell counts in indicated groups (*n* = 8, two‐way ANOVA followed by Tukey post hoc test). (h) IF staining images for periostin response to *siPdgfra* or/and *siPdgfrb* treatment, with and without TGF‐*β* (10 ng/mL) and D‐gal (200 µg/mL) stimulation for 24 h. Scale bar: 20 µm. (i) Western blot showing expression of the specific knockdown efficiency of PDGFRa and PDGFRb, CD34 and fibrosis‐related proteins in in the indicated groups. GAPDH served as a loading control. Quantifications shown in Figure S10l. Data are presented as the mean ± SEM.

### 2.6 PDGFR–JNK–JUNB Signaling Axis Is Required for CD34^+^ VICs Transdifferentiation Into Myofibroblasts During Ageing‐related AV Remodeling

To elucidate the downstream molecular mechanisms by which PDGFR signaling regulates CD34^+^ prog‐VIC activation, the mitogen‐activated protein kinase (MAPK) signaling pathways were examined. Pseudotime trajectory analysis of human VICs scRNA‐seq showed consistent and sustained upregulation of the JNK signaling pathway along the predicted developmental trajectory (Figure 7a). To investigate the intracellular mechanisms underlying CD34^+^ prog‐VIC activation, western blot analysis was performed for cells subjected to a time‐course treatment with both TGF‐*β* and D‐gal, with results revealing that the dual stimulation significantly promotes the myofibroblastic transdifferentiation of CD34^+^ prog‐VICs, as evidenced by the time‐dependent progressive upregulation of periostin (Figure 7b,c). Increased phosphorylation levels were also observed for JNK (p‐JNK), whereas ERK and p38 activation remained relatively unchanged (Figure 7b,c and Figure S11a). Furthermore, the siRNA‐mediated knockdown of *Pdgfra*, *Pdgfrb*, or both was performed in primary CD34^+^ VICs, and the knockdown of either receptor significantly reduced the phosphorylation of JNK (p‐JNK) (Figure 7d and Figure S11b). The JNK‐specific inhibitor SP600125 was used to verify the functional necessity of this axis, with pre‐treatment with SP600125 effectively blunting the TGF‐*β*/D‐gal‐induced upregulation of p‐JNK and the myofibroblast marker periostin (Figure 7e,f). By intersecting candidate transcription factors (TFs) predicted to bind to the *Periostin* promoter (using the TRRUST database) with TFs consistently expressed in human VICs (derived from our scRNA‐seq and bulk‐seq datasets) (Figure 7g), JUNB, a known downstream target of JNK signalling, was identified as a primary candidate (Figure 7g). A dual‐luciferase reporter system was then used to validate the functional interaction between JUNB and the *Periostin* promoter results confirming that JUNB significantly enhanced the luciferase activity at the *Periostin* promoter region (Figure 7i). Furthermore, the overexpression of *JUNB* markedly increased the *Periostin* mRNA transcription levels in 293T cells (Figure 7j). Knockdown of *JUNB* significantly inhibited TGF‐*β*/D‐gal‐induced transdifferentiation of CD34^+^ cells into myo‐VICs, downregulating key markers including Periostin, α‐SMA, collagen I, and FN1 (Figure 7k and Figure S11c), Conversely, *JUNB* overexpression effectively promoted this myofibrogenic transition, as evidenced by the corresponding upregulation of these myo‐VIC markers (Figure 7l and Figure S11d). Moreover, *JUNB* overexpression enhanced EdU incorporation, Transwell migration, and collagen gel contraction in primary CD34^+^ VICs, whereas *JUNB* silencing consistently attenuated cell proliferation, migration, and matrix contractility under TGF‐β/D‐gal stimulation (Figure S11e,f). Together, these molecular and functional findings establish JUNB as a key regulator of CD34^+^ VIC transdifferentiation into myo‐VICs. Consistent with these functional findings, pseudo‐time trajectory analysis of scRNA‐seq data revealed that *JUNB* expression levels were upregulated during the mid‐to‐late stages of prog‐VIC transdifferentiation, a temporal pattern that closely mirrored the expression dynamics observed for *Pdgfra* and *Pdgfrb* (Figure 7m). Furthermore, western blot analysis demonstrated that knocking down either *Pdgfra* or *Pdgfrb* resulted in consistent downregulation of the JUNB protein levels (Figure 7n and Figure S12a). To establish the intermediate signalling link, the JNK inhibitor SP600125 was utilized, which successfully abrogated the TGF‐*β*/D‐gal‐induced elevation of JUNB (Figure 7o and Figure S12b). More importantly, western blot analysis demonstrated that D‐gal, but not TGF‐β alone, significantly upregulated the expression of PDGFRa, PDGFRb, p‐JNK, and JUNB, with dual stimulation eliciting a synergistic engagement of the entire PDGFR‐JNK‐JUNB axis (Figure S12c). Collectively, these results provide robust evidence that the PDGFR–JNK–JUNBaxis serves as a vital regulatory cascade that dictates the fate of non‐BM CD34^+^ progenitors during age‐related valvular sclerosis (Figure 7p).

**FIGURE 7:**
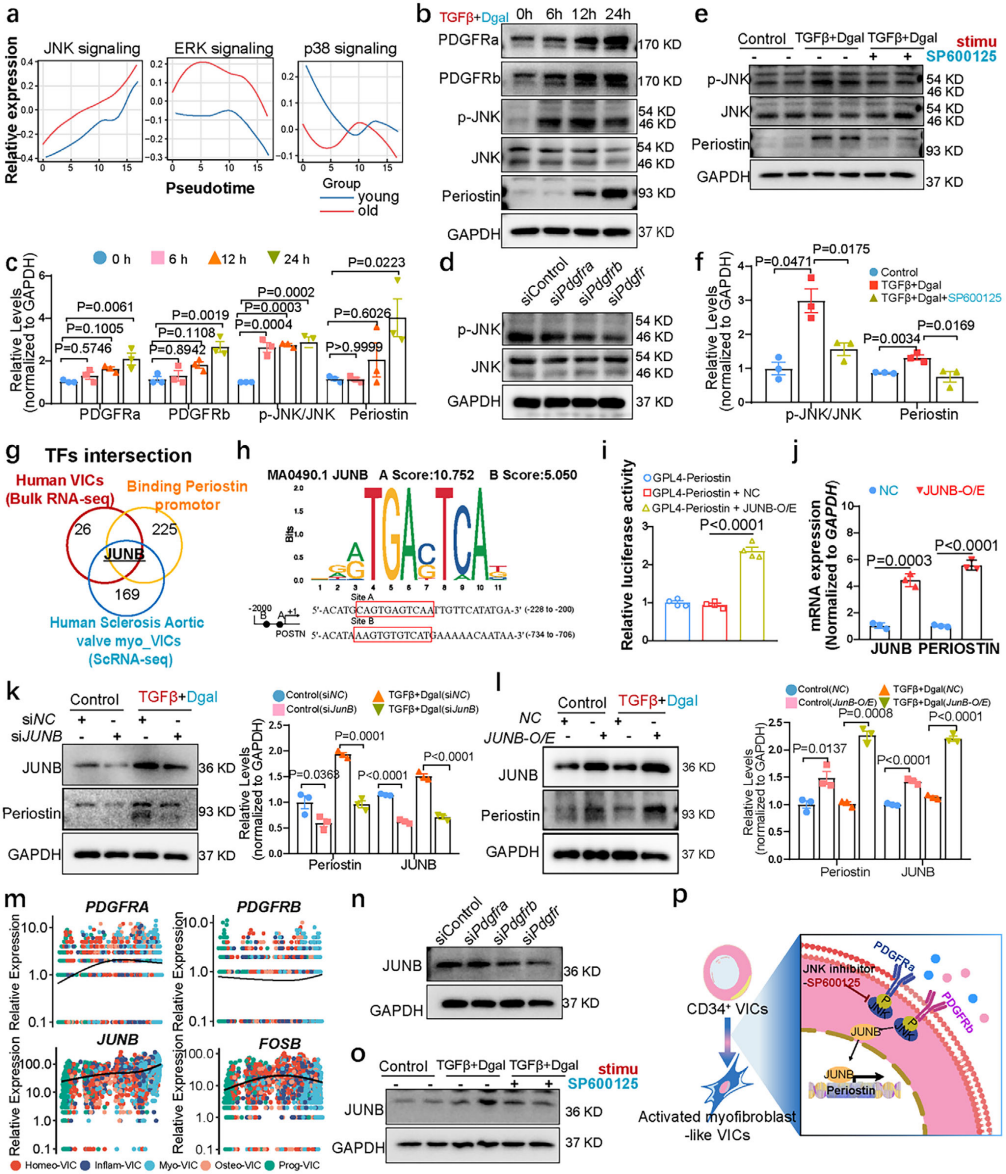
PDGFR‐JNK‐JUNB signalling axis is required for CD34^+^ cells transdifferentiation into myofibroblast‐like VICs during ageing‐related AV remodelling. (a) Curve diagrams displaying the relative expression levels of the MAPK signalling pathway in indicated human VICs (young and old group) along a pseudotime differentiation trajectory. (b) Western blot detection of PDGFRa, PDGFRb, p‐JNK, JNK, and periostin expression levels in cellular supernatant from primary CD34^+^ VICs, in response to TGF‐*β* (10 ng/mL) and D‐gal (200 µg/mL) treatment over indicated stimulation time. (c) Quantification of fold changes in the indicated protein expression of primary CD34^+^ VICs (*n* = 3, two‐way ANOVA followed by Tukey post hoc test). (d) Western blot showing the expression of p‐JNK and JNK proteins in response to *siPdgfra* or/and *siPdgfrb* treatment in primary CD34^+^ VICs cells. GAPDH served as a loading control. Quantifications shown in Figure S11b. (e,f) Western Blot analyses and quantifications of p‐JNK, JNK, and periostin changes in primary CD34^+^ VICs cells in response to TGF‐*β* (10 ng/mL) and D‐gal (200 µg/mL) stimulation, with or without Sp600125 (40 µmol/mL) treatment. (*n* = 3, two‐way ANOVA followed by Tukey post hoc test) (g) Veen plot showing the intersection of transcription factors (TFs) expressed by myo‐VICs or hVICs in human aortic valves, and TFs predicted to bind to periostin promoters in the JASPAR dataset. (h) Potential binding sites between periostin promoter and JUNB predicted using the JASPAR database. (i) Analysis of periostin promoter activity in the luciferase reporter assay of 293T cells, co‐transfected with the periostin promoter‐GPL‐basic plasmid and NC or JUNB plasmid (JunB‐O/E) (*n* = 4, two‐way ANOVA followed with Tukey's post hoc analysis). (j) Quantitative PCR analyses of periostin and JUNB in 293T cells, transfected with negative control (NC) or JUNB plasmid (JunB‐O/E) (*n* = 3, unpaired two‐tailed Student's t‐test). (k) Western Blot analyses showing quantifications of JUNB and periostin expression in primary CD34^+^ VICs transfected with small interfering RNA negative control (si*NC*) or si*JUNB*, with or without TGF‐*β* (10 ng/mL) and D‐gal (200 µg/mL) treatment (*n* = 3, unpaired two‐tailed Student's t‐test). (l) Representative western blot image showing JUNB and periostin expression levels in primary CD34^+^ VICs, transfected with negative control (NC) or JUNB plasmid (JUNB‐O/E). Quantification of the indicated protein levels are normalized to GAPDH (*n* = 3, unpaired two‐tailed Student's t‐test). (m) Gene expression trends for indicated genes across the pseudotime trajectory. (n) Western blot showing the expression of JUNB protein in response to *siPdgfra* or/and *siPdgfrb* treatment in primary CD34^+^ VICs cells; Quantifications are shown in Figure S12a. (o) Western Blot analyses of JUNB changes in primary CD34^+^ VICs cells in response to TGF‐*β* (10 ng/mL) and D‐gal (200 µg/mL) stimulation with or without Sp600125 (40 µmol/mL) treatment; Quantifications shown in Figure S12b. (p) Schematic diagram summarizing the molecular mechanism of CD34^+^ VICs activation and transdifferentiation into myofibroblast‐like VICs. Data are presented as the mean ± SEM.

## 3 Discussion

This study provides compelling evidence that non‐BM CD34^+^ prog‐VICs function as a fibroblast progenitor population participating in fibrotic remodeling in age‐associated AVS. By integrating human scRNA‐seq with natural ageing and D‐gal‐induced murine models, significant age‐dependent depletion of the CD34^+^ progenitor niche was characterized. Furthermore, using mouse lineage tracing and BMT models, the differentiation of non‐BM CD34^+^ prog‐VICs into periostin^+^ myo‐VICs was revealed. Functional assays such as the targeted ablation of systemic CD34^+^ cells, non‐BM CD34^+^ cells, and the CD34^+^Periostin^+^ transitional subpopulation demonstrated that these progenitors promote aortic valvular fibrosis, thickness, and stiffness in the D‐gal‐induced senescence or natural ageing model. Mechanistically, specific deletion of PDGFRa or PDGFRb in CD34^+^ cells in vivo significantly alleviated valvular pathological alterations. Moreover, the PDGFR–JNK–JUNB signaling axis was identified as an indispensable molecular switch that governs the pathological transdifferentiation of prog‐VICs into Periostin^+^ myo‐VICs.

### 3.1 Multidimensional Heterogeneity of CD34^+^ Population

The application of scRNA‐seq and high‐resolution lineage tracing has redefined our understanding of CD34^+^ cell heterogeneity within the aortic valve, mirroring findings in other cardiovascular compartments [23, 24]. Our study delineated this heterogeneity across two primary dimensions: lineage plasticity and cellular origin. Under physiological conditions, the CD34^+^ population comprises a diverse pool of cells, including a predominant cluster of fibroblast‐like VICs, a subset of endothelial cells, and sparse inflammatory cells. Paruchuri et al. previously demonstrated that individual valvular clones possess bipotential plasticity, adopting either an endothelial phenotype in response to VEGF‐A or a mesenchymal fate when exposed to TGF‐*β* [25]. This inherent plasticity is likely rooted in the observed progenitor‐level heterogeneity. Age‐associated AV remodeling was associated with a significant decline in the proportion of the CD34‐high prog‐VIC subpopulation in both human and mice specimens, coupled with an enhanced propensity for pathological transdifferentiation into matrix‐producing myo‐VICs. Our in vivo lineage tracing of the CD34^+^ cell lineage provides definitive evidence that these non‐BM progenitors serve as important sources of periostin^+^ myo‐VICs, driving the progression of fibrotic remodeling. Crucially, selective depletion of either CD34^+^ cells or the CD34^+^Periostin^+^ subpopulation significantly attenuates age‐related leaflet thickening, collagen deposition, and valvular stiffening. Lineage tracing of the CD34^+^ EPC compartment revealed no significant expansion of CD34‐derived ECs during protracted ageing. Although this appears to diverge from the established role of CD34^+^ EPCs in re‐endothelialisation, it likely reflects the temporal dynamics of the disease. Endothelial injury represents an early triggering event, whereas this study captures the chronic, cross‐sectional outcomes of valvular sclerosis, in which the reparative capacity of EPCs may have been exhausted or bypassed by the time significant fibrotic remodelling occurs. Further studies using acute endothelial injury models are warranted to refine these temporal distinctions.

The second layer of heterogeneity involves the distinct contributions of non‐BM versus BM CD34^+^ cells. Our BMT data demonstrated that the periostin^+^ myofibroblast pool is primarily replenished by non‐BM‐derived CD34^+^ prog‐VICs. Although these non‐BM cells are likely tissue‐resident progenitors, the potential contribution of non‐BM circulating progenitors cannot be entirely excluded without further parabiotic or local labelling studies. In contrast, the BM‐derived CD34^+^ fraction appeared to be largely restricted to the hematopoietic lineage, giving rise to inflammatory cells that infiltrate the valvular interstitium following injury. This functional segregation highlights the complexity of the CD34^+^ niche in AVS.

### 3.2 Ageing and AV Fibrotic Remodeling

The activation of VICs and their subsequent myofibroblastic or osteoblastic differentiation is a multifaceted process orchestrated by ageing, dyslipidaemia, disordered mineral metabolism, and chronic inflammation [26, 27]. Despite the well‐established link between cholesterol and cardiovascular disease, clinical trials targeting low‐density lipoprotein cholesterol (LDL‐C) reduction have yielded disappointing results in halting CAVD progression [28, 29, 30]. Departing from the metabolic‐centric HFD framework, which focuses on hyperlipidemia‐induced terminal calcification [31], this study used ageing‐focused models to address the early fibrotic phase of valvular disease. We demonstrated that ageing is in itself a sufficient and independent driver for valvular myofibrogenesis. This is consistent with emerging evidence that the loss of anti‐ageing genes or the accumulation of senescent VICs can directly trigger valvular dysfunction and disordered ECM deposition [32, 33, 34]. By shifting the focus from metabolic stress to cellular senescence, we found that ageing disrupted the quiescence of the non‐BM CD34^+^ prog‐VIC niche, promoting its pathological transition into periostin^+^ myo‐VICs.

### 3.3 PDGFR–JNK–JUNB Axis and AV Fibrosis

The PDGF/PDGFR system is a well‐established orchestrator of fibrotic responses across multiple organs, including the kidney, heart, skin, and lungs, signaling through its dimeric ligands and two distinct receptors, PDGFR‐*α* and PDGFR‐β [35]. Abundant evidence from myocardial infarction [36] and pressure‐overload models [37] has highlighted the potent fibrogenic effects of PDGF ligands, which are often mediated through the direct stimulation of fibroblast activation [38] and the synergistic up‐regulation of TGF‐*β* [39]. Consistent with these established paradigms, our in vivo functional assays demonstrated that lineage‐specific deletion of either *Pdgfra* or *Pdgfrb* within CD34^+^ cells significantly attenuated age‐related aortic leaflet thickening and fibrotic remodeling. This genetic evidence confirms that PDGFR signaling is a requisite driver in the pathological expansion of the myofibroblast pool. Furthermore, our data reveal a consistent molecular crosstalk [39] wherein dual stimulation with TGF‐*β* and D‐gal synergistically augments PDGFR expression. This interaction creates a self‐reinforcing fibrotic loop that is particularly hyperactive within the senescent valvular microenvironment. Specifically, senescence stress serves as a crucial priming event that synergizes with profibrotic signaling to fully amplify the PDGFR–JNK–JUNB cascade.

The MAPK signaling pathway is a well‐documented downstream effector of PDGFR that translates membrane receptor activation into genomic response [40, 41]. In this study, a combination of bioinformatics screening, western blot analysis, and transcription factor intersection assays was used to identify the JNK–JUNB axis as a specific intracellular engine driving the fibrotic fate of CD34^+^ prog‐VICs. In line with our data, both JNK [42] and JUNB [43, 44] have been reported as profibrotic mediators under various pathological conditions. Moreover, previous studies have established that JUNB binds directly to the proximal promoter of *Col1a2*, facilitating enhancer–promoter coalescence and driving collagen synthesis during tissue scarring [45]. Consistent with this paradigm, dual‐luciferase reporter assays confirmed that JUNB directly targets the periostin promoter in CD34^+^ prog‐VICs. While the established JUNB–COL1A2 axis primarily mediates deposition of the structural matrix, our findings suggest that the JUNB–POSTN interaction is a critical event leading to the initiation of the myofibrogenic program in the aged aortic valve. These findings uncovered the PDGFR–JNK–JUNB axis as a critical “molecular switch” that governs the pathological exit of CD34^+^ prog‐VICs from their homeostatic niche.

However, the potential contribution of non‐BM circulating CD34^+^ progenitors, which may be recruited from other systemic niches, remains unclear. Future studies employing parabiosis (coupled with BMT or lineage tracing) will be instrumental in definitively capturing the dynamic interplay between true tissue‐resident progenitors and their non‐BM systemic counterparts during valvular remodeling. While our in vivo data underscore that both PDGFR*α* and PDGFR*β* are essential for the myofibrotic activation of CD34^+^ cells, we were unable to fully dissect the distinct or synergistic contributions of these two receptors. Given their different ligand affinities and downstream biases in other tissues, it is plausible that PDGFR*α* and PDGFR*β* orchestrate complementary aspects of the fibrotic response in the aortic valve, which warrants further investigation.

In conclusion, non‐BM‐derived CD34^+^ prog‐VICs were identified as a crucial source of myofibroblasts in age‐associated AVS. By delineating the PDGFR–JNK–JUNB signaling axis, we provide a novel framework for early intervention to preserve valvular progenitor identity and prevent the progression of fibrotic remodeling.

## 4 Materials and Methods

### 4.1 Human AV Samples Collection

Nonsclerotic AV tissue samples were collected from young patients who had undergone surgery for aortic dissection and regurgitation, with the obtained aortic valves morphologically normal without obvious calcification or thickening. AV samples with sclerosis or stenosis were also obtained from patients who underwent AV replacement surgery. The baseline clinical characteristics of the study groups are listed in Table S1. All research was approved by the local ethics committee (Institutional Review Board approval [2025 B]; IIT Ethics Approval No.0673 and KY2021PJ191) in accordance with the Declaration of Helsinki, and all patients or their parents provided informed consent for the collection of samples.

### 4.2 Histopathology and Immunohistochemistry (IHC)

Gross bright‐field images of the aortic valves were obtained using an Olympus microscope (SZX16). To obtain the images, AV specimens were preserved in 4% paraformaldehyde (Servicebio, G1101) overnight, then embedded in paraffin and sliced into 5 µm thick sections. Hematoxylin and eosin (H&E), Masson's trichrome (Masson), Movat, Picro‐Sirius Red (PSR), Oil Red O, and Alizarin Red S staining were performed according to the manufacturer's guidelines. The AV structure was visualized using H&E staining; collagen deposition and proteoglycans in the AV were visualized using Masson and Movat staining; calcium deposition analysis was performed using Alizarin Red staining; and Oil Red O staining was used to examine atherosclerotic lesions in the AV. Embedded tissue slices were then subjected to standard dewaxing and rehydration, followed by 15‐min antigen retrieval with sodium citrate buffer, then incubated overnight at 4°C with the primary antibodies (CD34, Abcam, ab81829, 1:100), followed by the biotinylated secondary antibodies (Beyotime Biotechnology, A0192, 1:50). DAB substrate solution was used as a chromogen to visualize expression, with a hematoxylin solution used for counterstaining, with dehydration performed using progressively higher concentrations of ethanol and xylene before mounting in a neutral medium. The negative control for IHC was obtained by incubating with the secondary antibody only. Image‐Pro Plus software (version 6.0) was used to analyse the integrated optical density (IOD) by quantifying the brown‐stained CD34‐positive areas. The investigators performing the AV measurements were blinded to the experimental groups.

### 4.3 Primary Human Valvular Interstitial Cells (HVICs) Validation and Stimulation

HVICs were obtained from Saibaikang Biotechnology Co., Ltd. (Shanghai, China), with the primary HVIC culture system provided by iCell (Shanghai, China). Cells between passages 3 and 6 were used in the experiments. Cell lines were authenticated by immunofluorescent staining (Vimentin, Abcam, ab8978; aSMA, Sigma, F3777) and confirmed by CD31 (R&D Systems, AF3628) immunofluorescent staining. HVICs were subjected to 10 ng/mL of TGF*β* (Novoprotein, CK33) and 200 mg/mL of D‐gal (Sigma, G0750) to replicate the in vitro pro‐fibrotic environment associated with ageing.

### 4.4 Bulk RNA Sequencing

Total RNA extraction was performed on HVICs co‑treated with 10 ng/mL TGF‑*β* (Novoprotein, CK33) and 200 µg/mL D‑galactose (Sigma, G0750) for 48 h, with the RNA Nano 6000 Assay Kit employed to assess the RNA integrity. mRNA species with poly (A) tails were enriched using oligo(dT) magnetic beads, and following reverse transcription, cDNA was amplified and purified, followed by library construction and sequencing.

### 4.5 Mouse Generation and Breeding

Pdgfra*^flox/flox^* mice were obtained from GemPharmatech CO., Ltd, while *Cd34*‐CreER^T2^, *Postn*‐CreER^T2^, *Cd34*‐Dre knock‐in, Rosa26‐tdTomato, Rosa26‐DTR, Dou‐tdT‐DTR, and *Pdgfrb^flox/flox^* mice were purchased from Shanghai Model Organisms Center. Genomic DNA from mouse tail tissue was prepared by performing proteinase K lysis, precipitating with isopropanol, and washing with 70% ethanol before genotyping.

All animal procedures followed the guidelines set by the US National Institute of Health Guide for Care and Use of Laboratory Animals (eighth edition, 2011), and the study was approved by the Institutional Animal Care and Use Committee at Zhejiang University School of Medicine (No.2021/105).

### 4.6 AVS Animal Model

Mice of both sexes were randomly allocated to different experimental groups before housing in a standard feeding environment. The figure legends specify the number of mice used in each experiment. Two experimental models were utilized to mimic the degenerative AVS process: (1) a natural murine ageing model, for which mice were fed a regular diet, and those aged 2 months were classified as young and those aged 18–24 months were classified as old; (2) D‐gal induction in a murine model of accelerated ageing, for which groups were administered 100 mg/kg D‐gal via daily subcutaneous injections for approximately two months or 0.9% saline as a control.

Mice were euthanized using carbon dioxide (CO_2_) to minimize stress following the National Institutes of Health Guidelines for animal euthanasia, for which cohorts of 3–5 mice were allocated to an individual airtight chamber with a volume of 20 L, and CO_2_ gas with 99.99% purity was injected into the chamber at a flow rate of 10 L/min. All mice lost consciousness within 3 min of CO_2_ exposure, followed by the cessation of spontaneous respiration. To ensure thorough euthanasia, the CO_2_ flow was sustained for an additional minute. Each mouse was then meticulously examined for indicators of successful euthanasia, including the absence of respiratory activity, fading of eye pigmentation, and loss of pupillary light reflex. Following confirmation of euthanasia, the animals were promptly removed from the chamber, and the thoracic cavity surgically opened to access the heart. The hearts were then perfused via left ventricular puncture using PBS, and the aortic valves harvested and either placed in liquid nitrogen for Western Blot analysis or fixed with formalin for pathological staining and immunofluorescence detection.

### 4.7 Genetic Labelling, Conditional Knockout, and Depletion in Mice

Tamoxifen (Sigma, T5648) was dissolved in corn oil at a concentration of 20 mg/ml (Sigma, C8267) and administered via gavage every three days with a total of four doses (0.15 mg/g body weight) to label fluorescent protein in CD34^+^ cells from the *Cd34*‐CreER^T2^;R26‐tdT mice or CD34^+^Periostin^+^ cells from the *Cd34*‐Dre;*Postn*‐Cre ER^T2^;Dou‐tdT‐DTR mice, or to conditionally knockout *Pdgfra* or *Pdgfrb* in CD34^+^ cells from the *Cd34*‐CreER^T2^;*Pdgfra* ^fl/fl^ */Pdgfrb* ^fl/fl^ mice. To ablate CD34^+^ or CD34^+^Periostin^+^ cells in the Cd34‐CreER^T2^;R26‐DTR/tdTomato or *Cd34*‐Dre;*Postn*‐CreER^T2^;Dou‐tdT‐DTR mice, diphtheria toxin (DT, Sigma, D0564, 2 µg/ml) was administered intraperitoneally every other day at 20 ng/g body weight, with a total of five doses administered.

### 4.8 Bone Marrow Transplantation (BMT)

Recipient mice received a lethal irradiation dose of 9.0 Gy to induce complete myeloablation. Hematopoietic rescue was achieved by intravenous administration of 5 × 10^6^ bone marrow donor cells 6 h post‐irradiation. Following a 14‐day reconstitution window, successfully rescued chimeric mice were subjected to five consecutive daily pulses of tamoxifen over one week to label CD34^+^ cells with tdT, and/or five intraperitoneal injections of DT every other day to deplete tdT‑labelled CD34^+^ cells, followed by the administration of D‑galactose for disease modelling.

### 4.9 Mouse Echocardiography

To evaluate the heart and AV function, mice were anesthetized using 0.5%–1% Isoflurane and hair from the neck and chest area was removed with a depilatory cream to optimize the acoustic interface. Anaesthetized mice then placed on a heated blanket at 37°C and echocardiographic measurements taken using a VisualSonics Vevo 3100 ultrasound system, with B‐mode imaging used to obtain left ventricular parasternal short‐ and long‐axis views. The peak velocity of the AV was assessed from the suprasternal view using color and pulse‐wave Doppler, and cusp separation was measured with the inner‐edge‐to‐inner‐edge technique using the M‐Mode in the left parasternal long‐axis view. Echocardiographic measurements were also blinded.

### 4.10 Data Analyses for scRNA Sequencing

Data from the AV samples of three young and three elderly patients were obtained from the SRA repository (accession number: PRJNA562645) provided scRNA‐seq [46] and the pre‐processed count matrices were loaded into the Seurat R package (version 4.0.1) for downstream analyses.

Cells with < 300 or > 8500 identified genes were removed to exclude non‐cell entities or cell clusters, and those with over 10% mitochondrial reads were considered low quality and discarded. Doublets were then removed using the DoubletFinder R package (version 2.0.3), and after stringent quality control, a total of 60 374 cells (young: 32 846; old: 27 528) were retained for subsequent analysis.

### 4.11 Mouse AV CD34^+^ Cell Isolation, Cell Culture, and Differentiation

Aortic valve progenitor cells were isolated from mice according to a previously described protocol [47]. Briefly, minced valve tissue was plated onto T25 culture flasks pre‐coated with 0.1% gelatine (Byotime, C0317) and cultured in complete medium composed of DMEM (Sigma, D6429), 10% foetal bovine serum (HyClone, SH30396.03), 1% penicillin–streptomycin (Gibco, 15140122),1% N2 supplement (Invitrogen), 50 nm 2‐mercaptoethanol (Sigma), 2% B27 supplement (Invitrogen), and 20 ng/mL basic fibroblast growth factor (R&D). Cells were then passaged in series when reaching confluence following expansion from the explants. The primary cultures were maintained in complete medium that was changed every two days at a 1:3 split ratio, and CD34^−^ and CD34^+^ cells separated using a BD FACS ARIA II flow cytometer at passage 4 before placing them in DMEM containing 20% FBS. The sorted cells were then expanded for further experimentation.

For the differentiation assays, CD34^+^ cells were exposed to 10 ng/mL TGF‐*β* (Novoprotein, CK33) and 200 µg/mL D‐gal (Sigma, G0750) over 48 h to simulate in vivo fibrosis and senescence activation. For the inhibition experiments, cells were pretreated with 40 µM JNK inhibitor SP600125 (MCE, HY‐12041) over 24 h in the presence of TGF‑*β* and D‑galactose. Expression levels of fibrosis‑, extracellular matrix‑, and senescence‑ related proteins in CD34^+^ and/or CD34^−^ cells were assessed by western blot.

### 4.12 Transfection of siRNA and Plasmids

For the gene‐silencing experiments, siRNAs targeting *Pdgfra*, *Pdgfrb*, and *JunB*, along with their negative controls, were obtained from GenePharma, and siRNAs and siCtrl delivered into cells using the Lipofectamine RNAiMAX Transfection Reagent (Invitrogen, 13778075) under appropriate conditions. The siRNA sequences used in this study are listed in Table S3.

For the gene overexpression experiments, the JunB sequence was synthesized and inserted into the pcDNA3.1 vector (GenePharma) to generate the pcDNA3.1‐JunB overexpression plasmid. Both the recombinant plasmid and empty pcDNA3.1 vector were then transiently or stably introduced into 293T cells using Lipofectamine 3000 (Invitrogen) following the manufacturer's instructions, and cells harvested after 48 h for detection using PCR and western blotting.

### 4.13 Cell Transfection and Dual‐Luciferase Reporter System

Plasmids were transiently transfected into human embryonic kidney 293T cells using Lipofectamine 3000 (Invitrogen). To investigate the JunB‐periostin interaction, the GLP4‐basic vector containing the periostin promoter, control RL‐TK reporter plasmid, and either an empty vector (negative control) or a JunB‐expressing plasmid (pcDNA3.1(+); GenePharma) were co‐transfected into 293T cells. Cells were then lysed after 48 h, and firefly and Renilla luciferase activities were measured in sequence using the Dual‑Luciferase Reporter Assay System (Vazyme, DL101‑01) with a SpectraMax iD3 microplate reader.

### 4.14 Statistical Analysis

All experiments were performed blinded. Figures were prepared and statistical analyses were conducted using GraphPad Prism 9.0. Results are presented as the mean ± SEM. The numbers (n) represent the count of independent experiments or mice which were indicated in relevant figure legends. For comparisons between two groups, data with normal distribution were analyzed using an unpaired two‐tailed t‐test with Welch's correction for unequal variance, while data with non‐normal distribution were assessed using the nonparametric Mann–Whitney test. For comparisons involving more than two groups, normally distributed data were evaluated using one‐way ANOVA followed by Tukey's test for equal standard deviations (SDs), or by Brown‐Forsythe and Welch ANOVA tests with Tamhane's T2 test for unequal SDs. For cell culture data, normality and homogeneity of variance were tested prior to analysis. The Student's t‐test (unpaired t‐test) was employed for comparisons between two groups, whereas one‐way ANOVA, two‐way ANOVA, or two‐way mixed ANOVA, followed by an appropriate post hoc test (Tukey's post hoc test), was utilized for comparisons involving more than two groups. Statistical significance was set at *p* < 0.05.

More detailed descriptions of the materials and methods are provided in the Supplementary Information.

## Author Contributions

H.Y., J.C., Q.X., H.W., and X.Y. conceptualised and designed the study. G.R., Y.Z., P.Z., B.Z., H.W., and X.Y. performed most of the benchwork and data analyses. H.W. and X.Y. wrote the manuscript. H.Y., J.C. and Q.X. reviewed and edited the manuscript. All the authors have read and approved the final manuscript.

## Funding

This study was supported by the National Natural Science Foundation of China (82670486, 82604893, W2541023, 82400550, and 32171099), Ningbo Top Medical and Health Research Program (2025021121), the China Postdoctoral Science Foundation (2024M762029), the Ningbo Key Research and Development Program (2024Z232 and 2025Z178), and the Medical and Health Science and Technology Projects of Zhejiang Province (2024KY281 and 2025HY0872).

## Ethics Statement

Human Studies: The collection and use of human aortic valve tissue samples were approved by the local Ethics Committees (Institutional Review Board approval numbers: [2025B] IIT Ethics Approval No.0673 and KY2021PJ191). All procedures complied with the Declaration of Helsinki, and written informed consent was obtained from all patients or their legal guardians. Animal Studies: All animal protocols and experimental procedures were performed in accordance with the US National Institutes of Health Guide for the Care and Use of Laboratory Animals and were approved by the Institutional Animal Care and Use Committee of Zhejiang University School of Medicine (Approval No. 2021/105).

## Conflicts of Interest

The authors declare no conflicts of interest.

## Supporting information




**Supporting File 1**: advs78607‐sup‐0001‐SuppMat.pdf.

**Supporting File 2**: advs78607‐sup‐0002‐S1.pdf.

**Supporting File 3**: advs78607‐sup‐0003‐S2.pdf.

**Supporting File 4**: advs78607‐sup‐0004‐S3.pdf.

## Data Availability

All supporting data are available from the corresponding author upon reasonable request.

## References

[1] S. Kraler , M. C. Blaser , E. Aikawa , G. G. Camici , and T. F. Lüscher , “Calcific Aortic Valve Disease: From Molecular and Cellular Mechanisms to Medical Therapy,” European Heart Journal 43, no. 7 (2022): 683–697. 10.1093/eurheartj/ehab757 PMC8843796 34849696

[2] G. M. Novaro , R. Katz , R. J. Aviles , et al., “Clinical Factors, But Not C‐Reactive Protein, Predict Progression of Calcific Aortic‐Valve Disease,” Journal of the American College of Cardiology 50, no. 20 (2007): 1992–1998, 10.1016/j.jacc.2007.07.064. 17996566

[3] S. Coffey , B. Cox , and M. J. Williams , “The Prevalence, Incidence, Progression, and Risks of Aortic Valve Sclerosis,” Journal of the American College of Cardiology 63, no. 25 (2014): 2852–2861, 10.1016/j.jacc.2014.04.018. 24814496

[4] B. R. Lindman , M.‐A. Clavel , P. Mathieu , et al., “Calcific Aortic Stenosis,” Nature Reviews Disease Primers 2, no. 1 (2016): 16006, 10.1038/nrdp.2016.6. PMC5127286 27188578

[5] P. R. Goody , M. R. Hosen , D. Christmann , et al., “Aortic Valve Stenosis,” Arteriosclerosis, Thrombosis, and Vascular Biology 40, no. 4 (2020): 885–900, 10.1161/atvbaha.119.313067. 32160774

[6] J. D. Miller , R. M. Weiss , K. M. Serrano , et al., “Lowering Plasma Cholesterol Levels Halts Progression of Aortic Valve Disease in Mice,” Circulation 119, no. 20 (2009): 2693–2701, 10.1161/circulationaha.108.834614. PMC2740986 19433756

[7] J. D. Miller , R. M. Weiss , and D. D. Heistad , “Calcific Aortic Valve Stenosis: Methods, Models, and Mechanisms,” Circulation Research 108, no. 11 (2011): 1392–1412, 10.1161/circresaha.110.234138. PMC3150727 21617136

[8] E. Rabkin , M. Aikawa , J. R. Stone , Y. Fukumoto , P. Libby , and F. J. Schoen , “Activated Interstitial Myofibroblasts Express Catabolic Enzymes and Mediate Matrix Remodeling in Myxomatous Heart Valves,” Circulation 104, no. 21 (2001): 2525–2532, 10.1161/hc4601.099489. 11714645

[9] D. A. Towler , C. M. Giachelli , M. Scatena , Y. Tintut , and L. L. Demer , “Calcific Aortopathy in Response to Aging and Injury,” Circulation 153, no. 10 (2026): 769–785, 10.1161/circulationaha.125.072393. PMC12965821 41802024

[10] F. Schlotter , A. Halu , S. Goto , et al., “Spatiotemporal Multi‐Omics Mapping Generates a Molecular Atlas of the Aortic Valve and Reveals Networks Driving Disease,” Circulation 138, no. 4 (2018): 377–393, 10.1161/circulationaha.117.032291. PMC6160370 29588317

[11] J. L. Decano , Y. Iwamoto , S. Goto , et al., “A Disease‐driver Population Within Interstitial Cells of Human Calcific Aortic Valves Identified via Single‐Cell and Proteomic Profiling,” Cell Reports 39, no. 2 (2022): 110685, 10.1016/j.celrep.2022.110685. 35417712

[12] J. Wylie‐Sears , E. Aikawa , R. A. Levine , J.‐H. Yang , and J. Bischoff , “Mitral Valve Endothelial Cells With Osteogenic Differentiation Potential,” Arteriosclerosis, Thrombosis, and Vascular Biology 31, no. 3 (2011): 598–607, 10.1161/atvbaha.110.216184. PMC3210435 21164078

[13] I. F. Hall , E. Aikawa , J. Sluimer , A. H. Baker , and J. C. Kovacic , “Endothelial to Mesenchymal Transition in Cardiovascular Diseases: Molecular Insights and Clinical Perspectives,” European Heart Journal 47, no. 10 (2026): 1144–1158, 10.1093/eurheartj/ehaf670. PMC13016786 41128016

[14] D. Skowasch , S. Schrempf , N. Wernert , et al., “Cells of Primarily Extravalvular Origin in Degenerative Aortic Valves and Bioprostheses,” European Heart Journal 26, no. 23 (2005): 2576–2580, 10.1093/eurheartj/ehi458. 16115807

[15] T. B. Marvasti , F. J. Alibhai , R. D. Weisel , et al., “CD34(+) Stem Cells: Promising Roles in Cardiac Repair and Regeneration,” Canadian Journal of Cardiology 35, no. 10 (2019): 1311, 10.1016/j.cjca.2019.05.037. 31601413

[16] L. Jiang , T. Chen , S. Sun , et al., “Nonbone Marrow CD34 + Cells Are Crucial for Endothelial Repair of Injured Artery,” Circulation Research 129, no. 8 (2021): 146, 10.1161/circresaha.121.319494. 34474592

[17] X. Sun , T. Wang , H. Gong , et al., “Circulating CD34 + Fibroblast Progenitors Engaged in Heart Fibrosis of Allograft,” Circulation Research 138, no. 4 (2026): 326558, 10.1161/circresaha.125.326558. 41532318

[18] L. Du , X. Sun , H. Gong , et al., “Single cell and Lineage Tracing Studies Reveal the Impact of CD34+ Cells on Myocardial Fibrosis During Heart Failure,” Stem Cell Research & Therapy 14, no. 1 (2023): 33, 10.1186/s13287-023-03256-0. PMC9942332 36805782

[19] Y. Song , J. Yang , T. Li , et al., “CD34 + Cell–Derived Fibroblast‐Macrophage Cross‐Talk Drives Limb Ischemia Recovery Through the OSM‐ANGPTL Signaling Axis,” Science Advances 9, no. 15 (2023): add2632, 10.1126/sciadv.add2632. PMC12488030 37043578

[20] G. J. Lis , A. Dubrowski , M. Lis , et al., “Identification of CD34+/PGDFRα+ Valve Interstitial Cells (VICs) in Human Aortic Valves: Association of Their Abundance, Morphology and Spatial Organization With Early Calcific Remodeling,” International Journal of Molecular Sciences 21, no. 17 (2020): 6330, 10.3390/ijms21176330. PMC7503258 32878299

[21] M. Han , Z. Liu , L. Liu , et al., “Dual Genetic Tracing Reveals a Unique Fibroblast Subpopulation Modulating Cardiac Fibrosis,” Nature Genetics 55, no. 4 (2023): 665–678, 10.1038/s41588-023-01337-7. 36959363

[22] B. M. Klinkhammer , J. Floege , and P. Boor , “PDGF in Organ Fibrosis,” Molecular Aspects of Medicine 62 (2018): 44–62, 10.1016/j.mam.2017.11.008. 29155002

[23] X. Pu , P. Zhu , X. Zhou , et al., “CD34+ Cell Atlas of Main Organs Implicates its Impact on Fibrosis,” Cellular and Molecular Life Sciences 79, no. 11 (2022): 576, 10.1007/s00018-022-04606-6. PMC11803001 36315271

[24] L. Du , X. Wang , Y. Guo , et al., “Altered Lipid Metabolism Promoting Cardiac Fibrosis is Mediated by CD34^+^ Cell‐Derived FABP4^+^ Fibroblasts,” Experimental & Molecular Medicine 56, no. 8 (2024): 1869–1886, 10.1038/s12276-024-01309-9. PMC11372182 39198543

[25] S. Paruchuri , J.‐H. Yang , E. Aikawa , et al., “Human Pulmonary Valve Progenitor Cells Exhibit Endothelial/Mesenchymal Plasticity in Response to Vascular Endothelial Growth Factor‐A and Transforming Growth Factor‐β 2,” Circulation Research 99, no. 8 (2006): 861–869, 10.1161/01.RES.0000245188.41002.2c. PMC2810464 16973908

[26] X. Qian , L. Xu , Y. Zheng , et al., “Senescence‐Associated Metabolic Alterations Aggravate Calcific Aortic Valve Disease,” European Heart Journal 47, no. 30 (2026): 4133–4153, 10.1093/eurheartj/ehag191. 41841768

[27] J. H. Chen and C. A. Simmons , “Cell–Matrix Interactions in the Pathobiology of Calcific Aortic Valve Disease,” Circulation Research 108, no. 12 (2011): 1510–1524, 10.1161/circresaha.110.234237. 21659654

[28] K. L. Chan , K. Teo , J. G. Dumesnil , A. Ni , and J. Tam , “Effect of Lipid Lowering With Rosuvastatin on Progression of Aortic Stenosis,” Circulation 121, no. 2 (2010): 306–314, 10.1161/circulationaha.109.900027. 20048204

[29] S. J. Cowell , D. E. Newby , R. J. Prescott , et al., “A Randomized Trial of Intensive Lipid‐lowering Therapy in Calcific Aortic Stenosis,” New England Journal of Medicine 352, no. 23 (2005): 2389–2397, 10.1056/NEJMoa043876. 15944423

[30] A. B. Rossebø , T. R. Pedersen , K. Boman , et al., “Intensive Lipid Lowering With Simvastatin and Ezetimibe in Aortic Stenosis,” New England Journal of Medicine 359, no. 13 (2008): 1343–1356, 10.1056/NEJMoa0804602. 18765433

[31] R. M. Weiss , M. Ohashi , J. D. Miller , S. G. Young , and D. D. Heistad , “Calcific Aortic Valve Stenosis in Old Hypercholesterolemic Mice,” Circulation 114, no. 19 (2006): 2065–2069, 10.1161/circulationaha.106.634139. 17075015

[32] J. Chen , Y. Lin , and Z. Sun , “Deficiency in the Anti‐Aging Gene Klotho Promotes Aortic Valve Fibrosis Through AMPK α‐Mediated Activation of RUNX 2,” Aging Cell 15, no. 5 (2016): 853–860, 10.1111/acel.12494. PMC5013020 27242197

[33] X. Liu , K. Wang , Q. Zheng , et al., “Schisandrol B Inhibits Calcification of Aortic Valve by Targeting p53 Related Inflammatory and Senescence,” Biomedicine & Pharmacotherapy 178 (2024): 117241, 10.1016/j.biopha.2024.117241. 39111082

[34] J. Chen , J. Fan , S. Wang , and Z. Sun , “Secreted Klotho Attenuates Inflammation‐Associated Aortic Valve Fibrosis in Senescence‐Accelerated Mice P1,” Hypertension 71, no. 5 (2018): 877–885, 10.1161/hypertensionaha.117.10560. PMC5897144 29581213

[35] J. H. W. Distler , A.‐H. Györfi , M. Ramanujam , M. L. Whitfield , M. Königshoff , and R. Lafyatis , “Shared and Distinct Mechanisms of Fibrosis,” Nature Reviews Rheumatology 15, no. 12 (2019): 705–730, 10.1038/s41584-019-0322-7. 31712723

[36] L. Alex , I. Tuleta , S. C. Hernandez , et al., “Cardiac Pericytes Acquire a Fibrogenic Phenotype and Contribute to Vascular Maturation After Myocardial Infarction,” Circulation 148, no. 11 (2023): 882–898, 10.1161/circulationaha.123.064155. PMC10527624 37350296

[37] T. Tao , L. Du , P. Teng , et al., “Stem Cell Antigen‐1^+^Cell‐Derived Fibroblasts are Crucial for Cardiac Fibrosis During Heart Failure,” Cellular and Molecular Life Sciences 80, no. 10 (2023): 300, 10.1007/s00018-023-04957-8. PMC11073062 37740736

[38] L. Yao , B. H. Rathnakar , H. R. Kwon , et al., “Temporal Control of PDGFRα Regulates the Fibroblast‐to‐Myofibroblast Transition in Wound Healing,” Cell Reports 40, no. 7 (2022): 111192, 10.1016/j.celrep.2022.111192. PMC9423027 35977484

[39] R. Tuuminen , A. I. Nyka?nen , R. Krebs , et al., “PDGF‐A, ‐C, and ‐D but Not PDGF‐B Increase TGF‐β1 and Chronic Rejection in Rat Cardiac Allografts,” Arteriosclerosis, Thrombosis, and Vascular Biology 29, no. 5 (2009): 691–698, 10.1161/atvbaha.108.178558. 19213942

[40] E. Panzhinskiy , W. M. Zawada , K. R. Stenmark , and M. Das , “Hypoxia Induces Unique Proliferative Response in Adventitial Fibroblasts by Activating PDGFβ Receptor‐JNK1 Signalling,” Cardiovascular Research 95, no. 3 (2012): 356–365, 10.1093/cvr/cvs194. PMC3400360 22735370

[41] J. B. Demoulin and A. Essaghir , “PDGF Receptor Signaling Networks in Normal and Cancer Cells,” Cytokine & Growth Factor Reviews 25, no. 3 (2014): 273–283, 10.1016/j.cytogfr.2014.03.003. 24703957

[42] N. Reich , M. Tomcik , P. Zerr , et al., “Jun N‐terminal Kinase as a Potential Molecular Target for Prevention and Treatment of Dermal Fibrosis,” Annals of the Rheumatic Diseases 71, no. 5 (2012): 737–745, 10.1136/annrheumdis-2011-200412. 22258492

[43] P. Maity , K. Singh , L. Krug , et al., “Persistent JunB Activation in Fibroblasts Disrupts Stem Cell Niche Interactions Enforcing Skin Aging,” Cell Reports 36, no. 9 (2021): 109634, 10.1016/j.celrep.2021.109634. 34469740

[44] M. A. Missinato , S. Murphy , M. Lynott , et al., “Conserved Transcription Factors Promote Cell Fate Stability and Restrict Reprogramming Potential in Differentiated Cells,” Nature Communications 14, no. 1 (2023): 1709, 10.1038/s41467-023-37256-8. PMC10043290 36973293

[45] M. Ponticos , C. Harvey , T. Ikeda , D. Abraham , and G. Bou‐Gharios , “JunB Mediates Enhancer/Promoter Activity of COL1A2 Following TGF‐β Induction,” Nucleic Acids Research 37, no. 16 (2009): 5378–5389, 10.1093/nar/gkp544. PMC2760791 19561194

[46] Y. Huang , C. Wang , T. Zhou , et al., “Lumican Promotes Calcific Aortic Valve Disease Through H3 Histone Lactylation,” European Heart Journal 45, no. 37 (2024): 3871–3885, 10.1093/eurheartj/ehae407. 38976370

[47] K. Chen , R. Mou , P. Zhu , et al., “The Effect of Lymphangiogenesis in Transplant Arteriosclerosis,” Circulation 147, no. 6 (2023): 482–497, 10.1161/circulationaha.122.060799. 36515099

