# Supplementary material for: CD34+ Progenitors Promote Ageing‐Associated Valve Sclerosis through the PDGFR–JNK–JUNB Pathway
Source: Adv Sci (Weinh). 2026 Nov 10:e78607. Online ahead of print. doi: 10.1002/advs.78607 (PMC13752233; doi:10.1002/advs.78607)
Supplement: Supplementary file 1 [file ADVS-9999-e78607-s001.pdf]

Table S2. Antibodies

Target antigen	Vendor or Source	Catalog #	Working concentration
tdTomato	Rockland	600-401-379	1:200
tdTomato	SICGEN	AB8181-200	1:200
CD31	R&D	AF3628	1:50
SMA-FITC	Sigma	F3777	1:300
CD34	Abcam	ab81829	1:100 for Immunofluorescence; 1:1000 for Western Blot
CD45	R&D	AF114	1:50
CD68	Abcam	ab125212	1:50 for immunofluorescence; 1:1000 for western blot
PDGFRa	R&D	AF1062	1:50 for immunofluorescence; 1:1000 for western blot
PDGFRb	R&D	AF1042	1:50 for immunofluorescence; 1:1000 for western blot
Vimentin	Abcam	Ab8978	1:50 for immunofluorescence; 1:1000 for western blot
Periostin	R&D	AF2955	1:50 for immunofluorescence; 1:1000 for western blot
donkey anti-rabbit IgG Alexa Fluor 555	Invitrogen	A-31572	1:500
donkey anti-rabbit IgG Alexa Fluor 647	Invitrogen	A-48272	1:500
donkey anti-goat IgG Alexa Fluor 555	Invitrogen	A-32816	1:500
donkey anti-rat IgG Alexa Fluor 647	Invitrogen	A-48272	1:500
donkey anti-mouse IgG Alexa Fluor 488	Invitrogen	A-11055	1:500
Rat IgG Isotype Control	Invitrogen	31933	1:500
Rabbit IgG Isotype Control	Invitrogen	02-6102	1:500
Mouse IgG Isotype Control	Invitrogen	31903	1:500
Goat IgG Isotype Control	Invitrogen	31245	1:500
Fibronectin (FN1)	Abcam	ab2413	1:1000
α- SMA	Abcam	ab21027	1:1000
Collagen I	Servicebio	GB115707	1:1000
p53	Abcam	ab26	1:1000
PCNA	Abcam	ab29	1:1000

p-JNK (Thr183/Tyr185)	Servicebio	GB15018	1:1000
JNK	diagbio	db2618	1:1000
p-p38 (Thr180/Tyr182)	Servicebio	GB153380	1:1000
p38	Servicebio	GB154685	1:1000
ERK	CST	4695	1:1000
p-ERK (Thr202/Tyr204)	CST	9101S	1:1000
JunB	Abcam	ab128878	1:1000
β-Actin	CST	4970	1:1000
HRP-conjugated GAPDH	Proteintech	HRP-60004	1:2000
p21	CST	2947	1:1000
p16	Zenbio	R23897	1:1000
