# Supplementary material for: CD34+ Progenitors Promote Ageing‐Associated Valve Sclerosis through the PDGFR–JNK–JUNB Pathway
Source: Adv Sci (Weinh). 2026 Nov 10:e78607. Online ahead of print. doi: 10.1002/advs.78607 (PMC13752233; doi:10.1002/advs.78607)
Supplement: Supplementary file 2 [file ADVS-9999-e78607-s002.pdf]

Supplementary Information

CD34[+] Progenitors Promote Ageing-associated Valve Sclerosis Through the PDGFR–JNK–JUNB Pathway

Xiaoping Yang[1#], Hong Wu[2#], Guoqiang Ren[1#] Yuesheng Zhang[3], Zuoshi Wen[3], Pengwei Zhu[3], Zhangquan Ying[3], Liujun Jiang[3], Tingting Tao[4], Bohuan Zhang[3], Linwen Zhu[5], Han Wang[6], Han Zhang[3], Qingbo Xu[3☒], Jun Cheng[6☒] and Honghua Ye[1☒]

1. Department of Cardiology, The Affiliated Lihuili Hospital of Ningbo University, Ningbo University, Ningbo, Zhejiang, China;
2. Department of Geriatrics, Xinhua Hospital, Shanghai Jiao Tong University School of Medicine, Shanghai, China;
3. Department of Cardiology, The First Affiliated Hospital, Zhejiang University School of Medicine, Hangzhou, China;
4. Department of Cardiothoracic Surgery, The First Affiliated Hospital, Zhejiang University School of Medicine, Hangzhou, China;
5. Ningbo Key Laboratory for Innovative Research in Aortic Surgery Diagnosis and Treatment, Department of Cardiothoracic Surgery, The Affiliated Lihuili Hospital of Ningbo University, Ningbo University, Ningbo, Zhejiang, China
6. Department of Vascular Surgery, Renji Hospital, Shanghai Jiao Tong University School of Medicine, China;
7. Key Laboratory of Medical Electrophysiology of the Ministry of Education and Medical Electrophysiological Key Laboratory of Sichuan Province, Collaborative Innovation Center for Prevention and Treatment of Cardiovascular Disease, Institute of Cardiovascular Research, Public Center of Experimental Technology, Haemodynamics and Medical Engineering Combination Key Laboratory of Luzhou, Southwest Medical University, Luzhou, China

First author

Xiaoping Yang

Telephone: +86-15158380820   E-mail: yzyangxiaoping@163.com

☒Address correspondence to:

Qingbo Xu, MD, PhD Department of Cardiology, The First Affiliated Hospital, Zhejiang University School of Medicine, Hangzhou, China, E-mail: qingbo_xu@zju.edu.cn

Or Jun Cheng, Key Laboratory of Medical Electrophysiology of the Ministry of Education and Institute of Cardiovascular Research, Southwest Medical University, 319 Zhongshan Road, Luzhou 646000, China Tel: +86 830-3160619, Email: lzcj1221@swmu.edu.comOr Honghua Ye, MD, The Affiliated Lihuili Hospital of Ningbo University, Health Science Center, Ningbo University, No.57, Xingning Road, Ningbo, Zhejiang Province, 315040, China, Email: honghuaye@nbu.edu.cn

Figures S1–S12

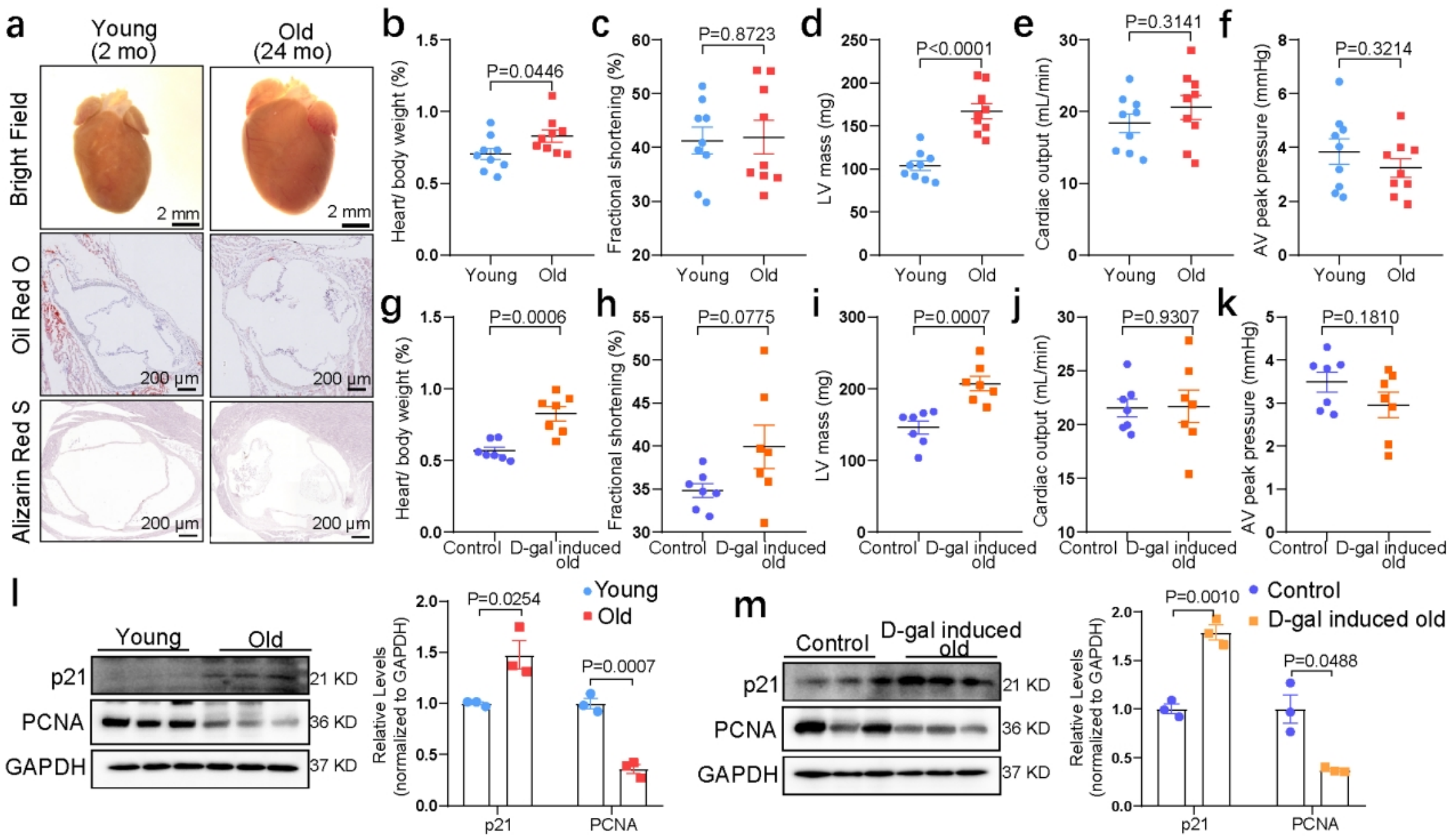

FIGURE S1 (Related to FIGURE 1) Senescence induced cardiac remodelling is indicated by an increase in LV hypertrophy. (a) Representative images of gross heart pathology, and Oil Red O and Alizarin Red S staining of aortic valve from young and old mice. Scale bars: 2 mm in bright field and 200 μm in pathological staining. (b–f) Heart/body weight ratio, fractional shortening fraction, LV mass, cardiac output, and AV peak pressure in young and old mice (n=9, unpaired two-tailed Student's t-test). (g–k) Heart/body weight ratio, fractional shortening fraction, LV mass, cardiac output, and AV peak pressure in control and D-galactose (D-Gal) induced old WT mice (n=7, unpaired two-tailed Student's t-test). (l) Western blotting of p21 and PCNA in aortic valves from young and old mice (n=3, unpaired two-tailed Student's t-test). (m) Western blotting of p21 and PCNA in AV from control and D-gal induced old WT

mice (n=3, unpaired two-tailed Student's t-test). Data are presented as the mean ± SEM. LV: left ventricular; AV: aortic valve.

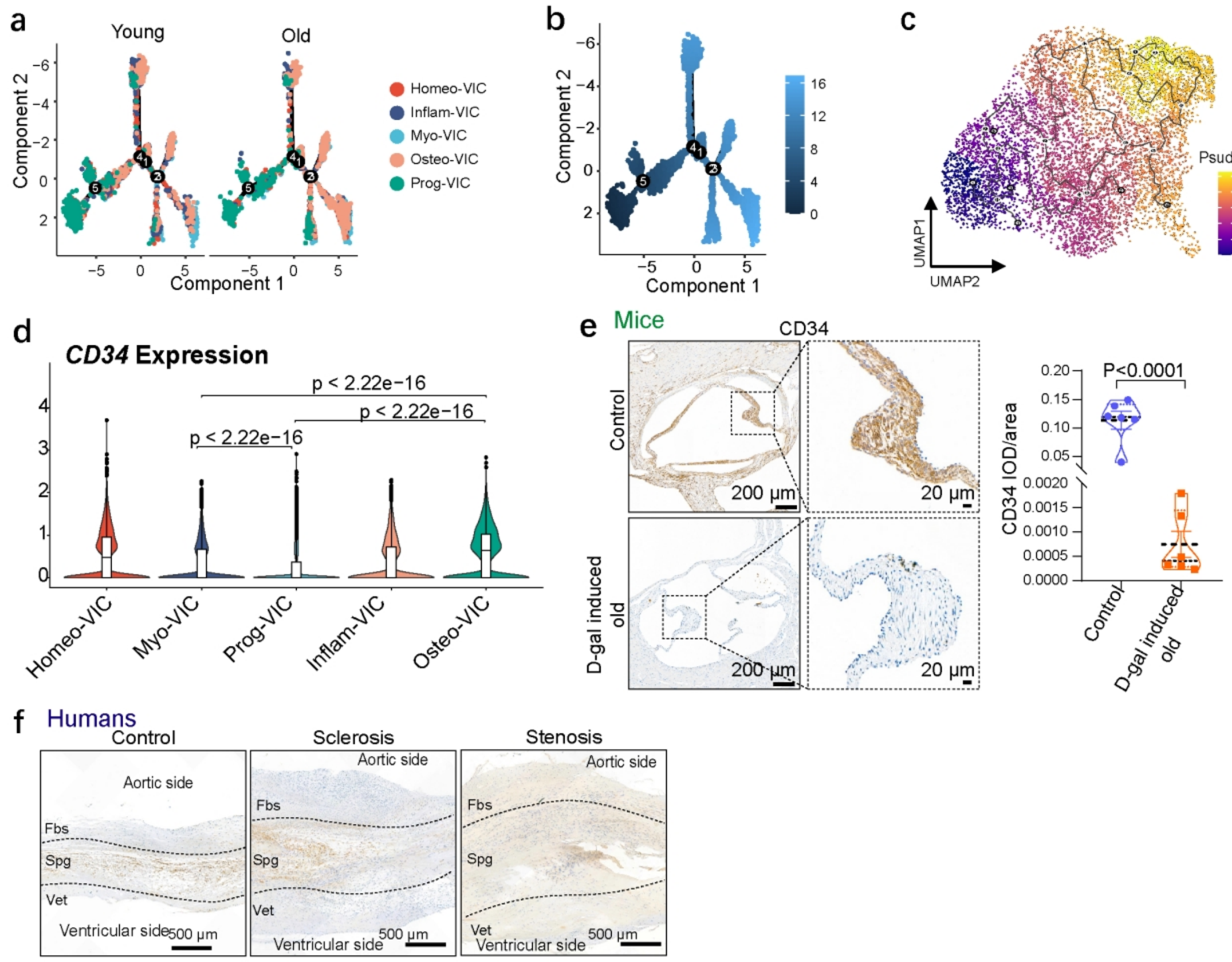

FIGURE S2 (Related to FIGURE 2). Single-cell sequencing indicates decreased CD34 expression in aortic valve sclerosis process. (a) Pseudotime trajectory coloured by VIC subcluster in each group. (b) Pseudotime trajectory analysis of all VICs in 2 groups, coloured by pseudotime. (c) Pseudotime trajectory suggesting a differentiation trend from Prog_VICs toward Myo_VICs during transition. (d) Violin plots showing *CD34* gene expression levels in the each VIC subcluster. Statistical *p* values from Wilcoxon testing are added between groups. (e) Representative immunohistochemical (IHC) images illustrating CD34 expression pattern observed for the aortic valve of control in D-gal induced old mice, accompanied by magnified views of the boxed regions. Dot plot (right) displays the average optical density of CD34-positive staining in the aortic valves of the different groups (n=6, unpaired two-tailed Student's t-test). Scale bars: 200 μm and 20 μm. (f) Representative IHC images illustrating CD34 expression pattern observed for human with sclerosis and stenosis, together with non-sclerotic and non-calcific AV tissues. Scale bars: 500 μm. Data are presented as the mean ± SEM.

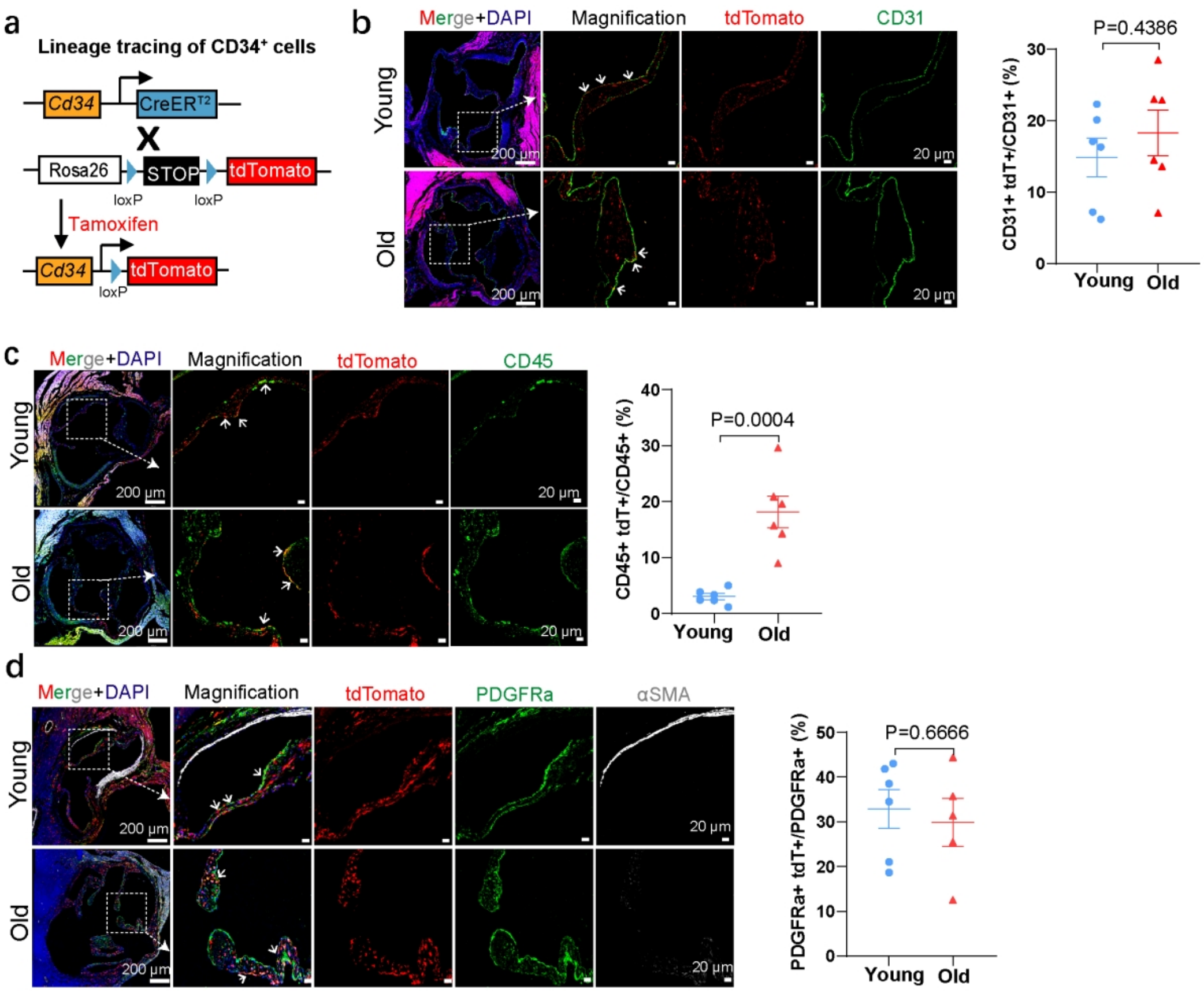

FIGURE S3 (Related to FIGURE 3) CD34⁺ cellular lineage tracing in aortic valve under different physiological conditions. (a) Schematic diagram illustrating the construction strategy of *Cd34*-CreER^{T2};R26-tdT mice. (b) Immunostaining of tdTomato and CD31 on aortic valve sections in young (2-mo) and old (24-mo) *Cd34*-CreER^{T2}; R26-tdT mice, with boxed region showing magnification (left), along with quantification of the percentages of CD31$^+$tdT$^+$ cells in the CD31$^+$ cells in the indicated group (n=6, unpaired two-tailed Student's t-test). Scale bars: 200 μm, and 20 μm in magnification images. (c) Immunostaining of tdTomato and CD45 on aortic valve sections in young (2-mo) and old (24-mo) *Cd34*-CreER^{T2}; R26-tdT mice, with boxed region showing magnification (left), along with quantification of the percentages of CD45$^+$ tdT$^+$ cells in the CD45$^+$ cells in the indicated group (n=6, unpaired two-tailed Student's t-test). Scale bars: 200 μm, and 20 μm in magnification images (d) Immunostaining of tdTomato, PDGFRa, and *α*SMA on aortic valve sections in young (2-mo) and old (24-mo) *Cd34*-CreER^{T2}; R26-tdT mice, with boxed region showing magnification (left), along with quantification of the percentages of PDGFRa$^+$ tdT$^+$ cells in the PDGFRa$^+$ cells in the indicated group (n=5-6, unpaired two-tailed Student's t-test). Scale bars: 200 μm, and 20 μm in magnification images. Data are presented as mean ± SEM.

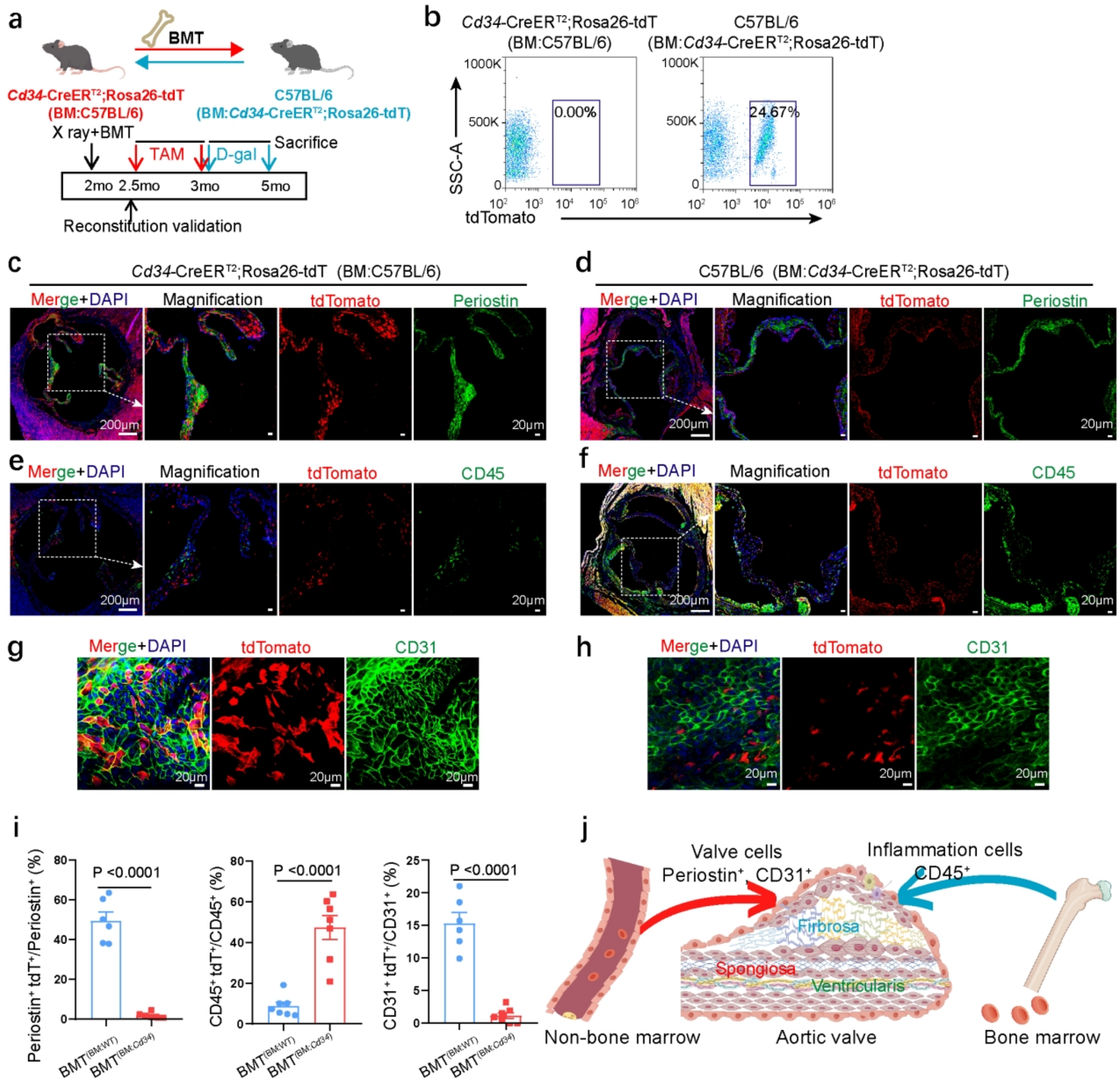

FIGURE S4 (Related to FIGURE 3) Non-BM derived CD34⁺ cells function as valvular interstitial progenitor cells. (a) Sketch of the experimental design used to generate two types of chimeric mice via bone marrow transplantation (BMT). (b) Flow cytometric analysis of tdTomato⁺ expression in bone marrow cells from indicated chimeric mice following TAM induction. (c) IF staining of tdTomato and periostin on aortic valve sections from $\mathrm{BMT}^{\mathrm{WT}\rightarrow Cd34\ (\mathrm{BM:\ WT})}$ chimeric mice subjected to 2 months of D-gal injection, with magnified boxed regions showing split channels to the right. Scale bars: 200 μm and 20 μm. (d) IF staining of tdTomato and periostin on aortic valve sections from $\mathrm{BMT}^{Cd34\rightarrow \mathrm{WT}(\mathrm{BM:}\ Cd34)}$ chimeric mice subjected to 2 months of D-gal injection, with magnified boxed regions showing split channels to the right. Scale bars: 200 μm and 20 μm. (e) IF staining of tdTomato and CD45 on aortic valve sections from $\mathrm{BMT}^{\mathrm{WT}\rightarrow Cd34\ (\mathrm{BM:\ WT})}$ chimeric mice subjected to 2 months of D-gal injection, with magnified boxed regions showing split channels to the right. Scale bars: 200 μm and 20 μm. (f) IF staining of tdTomato and CD45 on aortic valve sections from $\mathrm{BMT}^{Cd34\rightarrow \mathrm{WT}(\mathrm{BM:}\ Cd34)}$ chimeric mice subjected to 2 months of D-gal injection, with magnified boxed regions showing split channels to the right. Scale bars: 200 μm and 20 μm. (g) *En-face* immunostaining of tdTomato and CD31 on the centric aortic

valve endothelial layer of $BMT^{WT \rightarrow Cd34\ (BM:\ WT)}$ chimeric mice subjected to 2 months of D-gal injection. Scale bar: 20 μm. (h) *En-face* immunostaining of tdTomato and CD31 on the centric aortic valve endothelial layer of $BMT^{Cd34 \rightarrow WT (BM:\ Cd34)}$ chimeric mice subjected to 2 months of D-gal injection. Scale bar: 20 μm. (i) Quantification of the percentages of tdT^{+}/$periostin^{+}$ cells comprising the $periostin^{+}$ cells (left), tdT^{+}/$CD45^{+}$ cells in $CD45^{+}$ cells (medium), and tdT^{+}/$CD31^{+}$ cells comprising $CD31^{+}$ cells (right) in aortic valve tissues from two types of chimeric mice ($BMT^{BM:\ WT}$, $BMT^{BM:\ Cd34}$) injected with D-gal over 2 months (n=6-7, unpaired two-tailed Student's t-test). (j) Schematic diagram showing transdifferentiation fate for $CD34^{+}$ cells from different origins in D-gal induced mice. Data are presented as the mean ± SEM. WT: wild-type mice with a C57BL/6 background; Cd34: *Cd34*-CreER^{T2};R26-tdT mice.

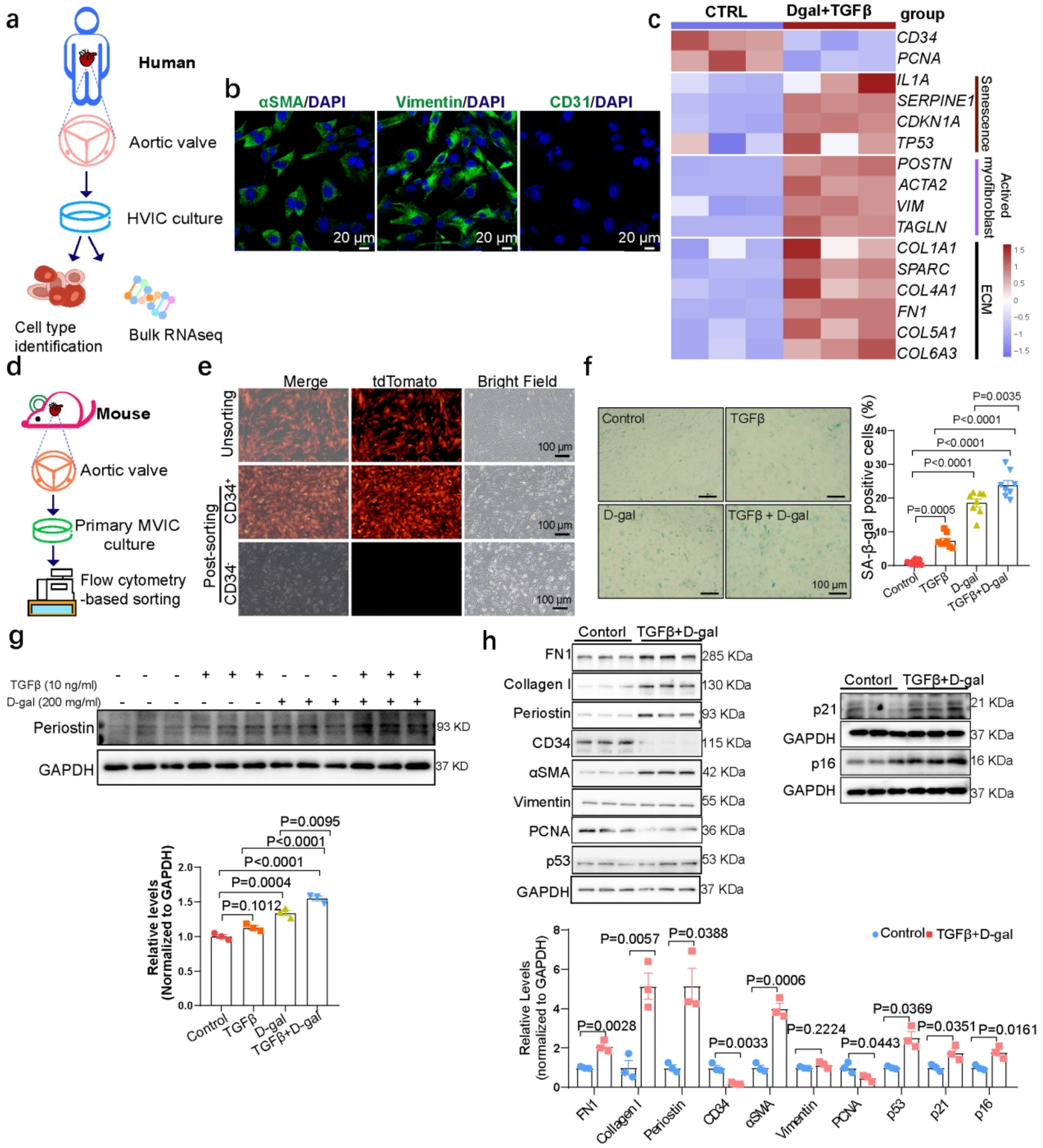

FIGURE S5 (Related to FIGURE 3) Establishment of the *in vitro* cellular

senescence model. (a) Schematic procedure used for HVICs identification and bulk RNA-seq analysis. (b) Immunofluorescence (IF) staining of *α*SMA, vimentin, and CD31 expression, used to identify cell type. Scale bars: 20 µm. (c) Heatmap showing differentially expressed genes (DEGs) in HVICs treated with TGF-*β* (10 ng/mL) and D-gal (200 µg/mL) over 48 h. Gene expression levels were normalised as log2-transformed counts per million (log2 CPM). (Note: All displayed genes reached statistical significance with an adjusted P < 0.05) (d) Schematic procedure showing isolation of aortic valvular interstitial cells from $CD34^+$ and $CD34^-$ mice. (e) Representative image showing $CD34^+$ aortic valvular interstitial cells from tamoxifen (Tam)-treated *Cd34*-CreER;R26-tdT mice before (unsorting) and after (post- sorting) flow cytometric sorting. (f) Senescence-associated *β*-Gal (SA-β-gal) staining assay showing cellular senescence in aortic valvular $CD34^+$ cells, followed by TGF-*β* (10 ng/mL) and/or D-gal (200 µg/mL) stimulation for 48h, SA-β-gal-positive cells are stained in blue. Scale bar: 100 µm. Dotplot (right) displaying the percentages of SA-β-gal-positive cells in primary valvular cells across different groups (n=8, one-way ANOVA followed by Tukey post hoc test). (g)Western blotting of Periostin in $CD34^+$ VICs, followed by TGF-*β* (10 ng/mL) and/or D-gal (200 µg/mL) stimulation for 12h (n=3, one-way ANOVA followed by Tukey post hoc test). (h) Western blot analyses show fibrosis- and senescence-related proteins following TGF-*β* (10 ng/mL) and D-gal (200 µg/mL) treatment for 48 h in mouse $CD34^+$ VICs (n = 3, unpaired two-tailed Student's t-test). Data are presented as the mean ± SEM.

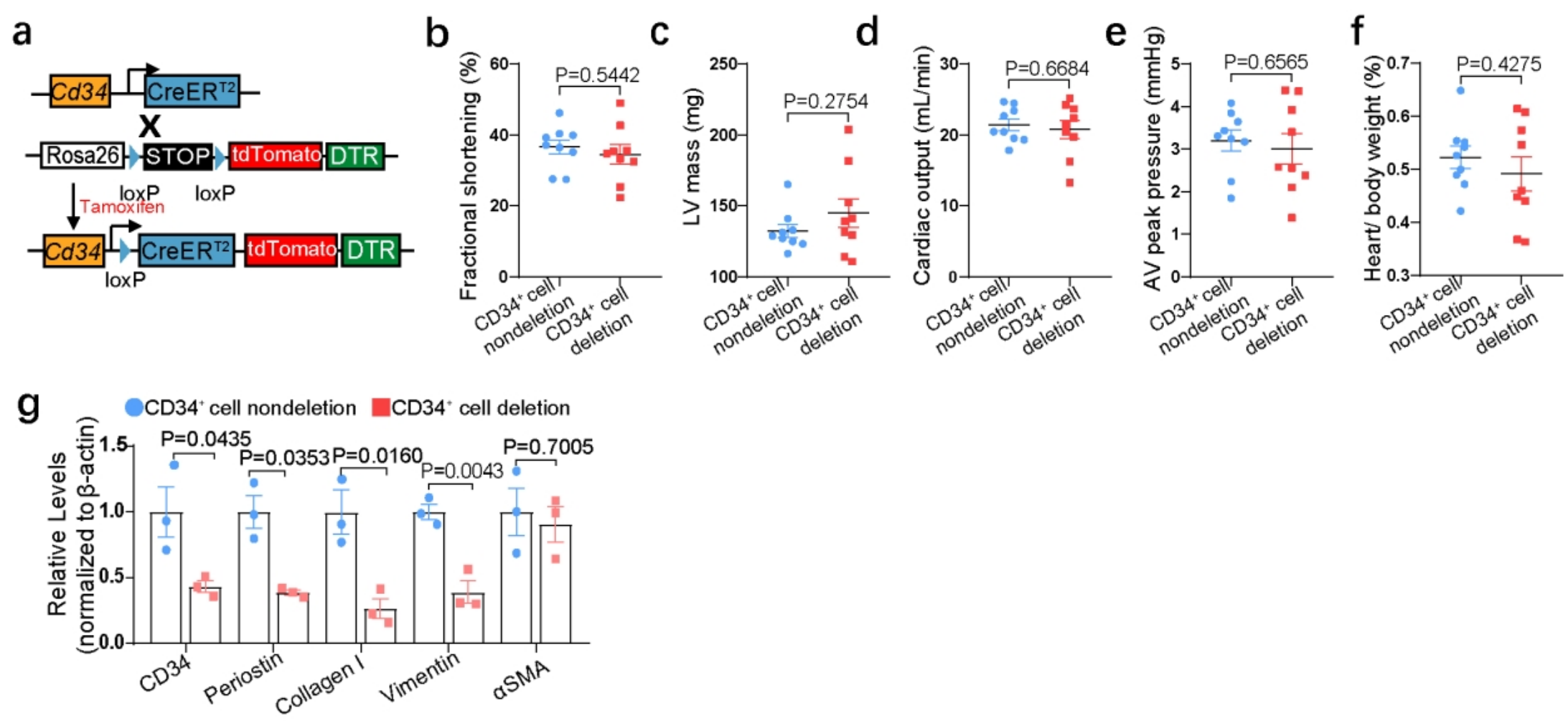

FIGURE S6 (Related to FIGURE 4) Deletion of $CD34^+$ cells show no changes in fractional shortening, LV mass, cardiac output, AV peak pressure and heart/ body weight. (a) Schematic diagram illustrating construction strategy for *Cd34*-$CreER^{T2}$;R26- tdTomato/DTR mice. (b–f) Fractional shortening fraction, LV mass, cardiac output, AV peak pressure, heart/body weight ratio in $CD34^+$ cell deletion group and non-deletion group after D-gal injection over 2 months (n=9, unpaired two-tailed Student's t-test). (g) Quantifications of CD34 and fibrosis-related proteins in the indicated groups (n=3, unpaired two-tailed Student's t-

test); Related to Figure 6i.

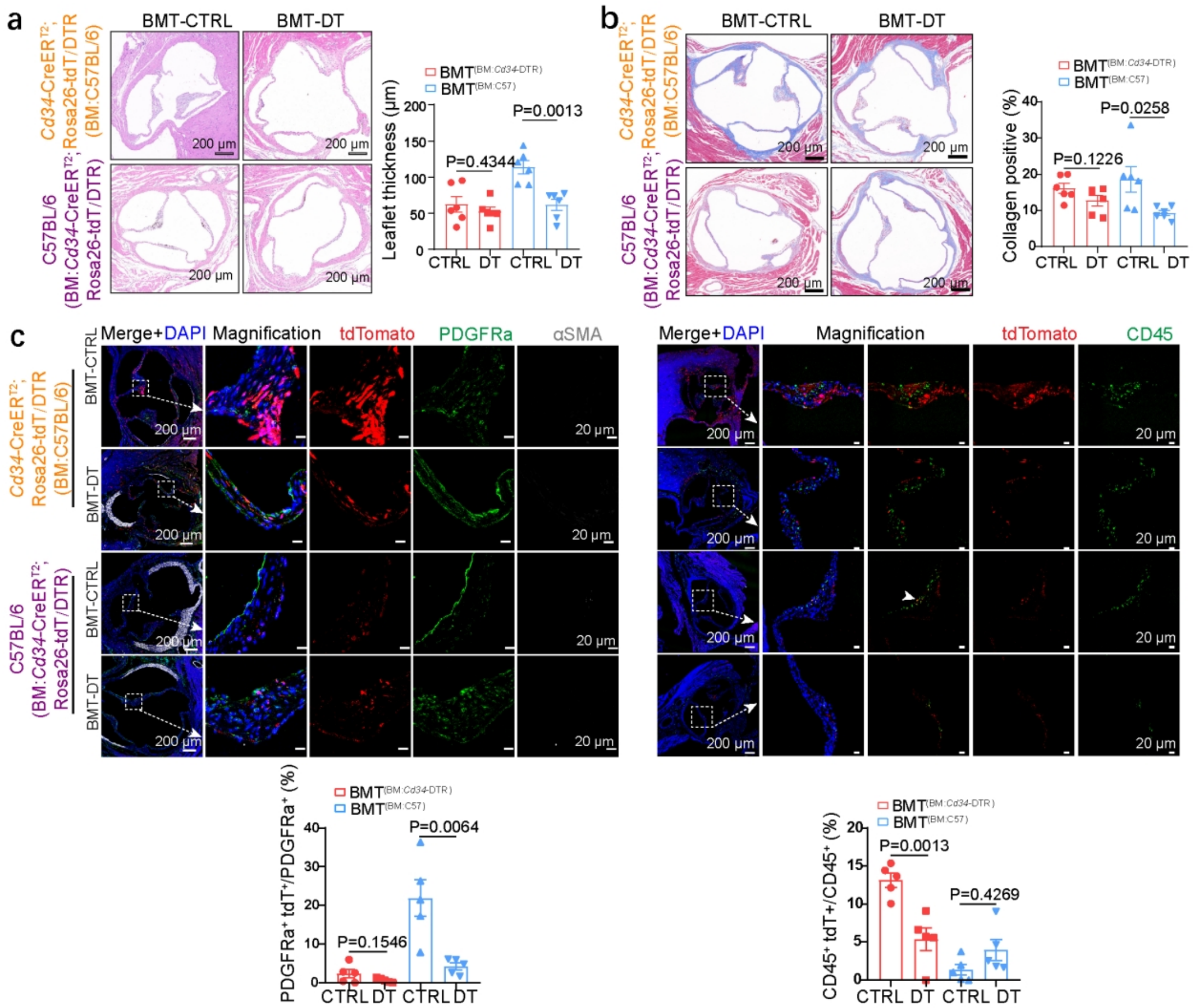

FIGURE S7 (Related to FIGURE 4) Deletion of non-BM CD34⁺ cells decreases the extent of aortic valve modelling. Representative images of aortic valves from indicated groups observed using HE **(a)** and Masson staining **(b)**. Scale bar: 200 μm. Quantification (right) of leaflet thickness (a) and aortic valve collagen positive fractions (b) in aortic valves in the indicated groups (n=6, unpaired two-tailed Student's t-test). **(c)** IF staining of aortic valve sections of indicated groups for PDGFRa, tdTomato, *α*SMA (left), tdTomato, and CD45 (right) following D-gal induction over 2 months. Box regions are magnified on the right with split channels (n=5, unpaired two-tailed Student's t-test). Scale bars: 200 μm and 20 μm. Data are presented as the mean ± SEM.

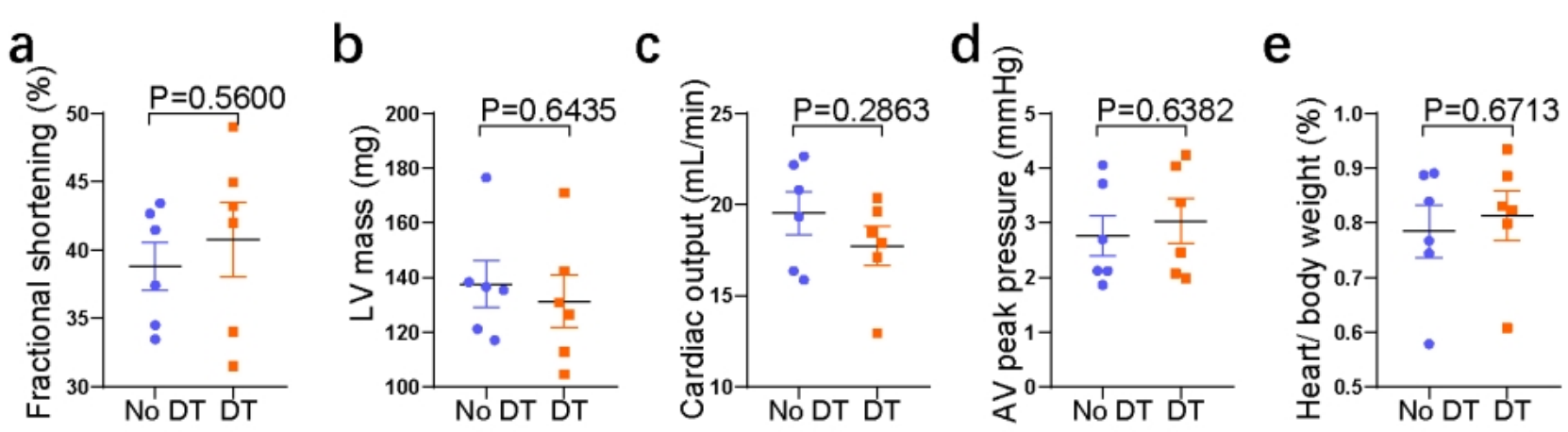

FIGURE S8 (Related to FGURE 5) *Cd34*-Dre;*Postn*-CreER^{T2}; Dou-tdT-DTR mice with DT injection show no changes in fractional shortening, LV mass,

cardiac output, AV peak pressure and heart/ body weight. (a) Fractional shortening fraction, (b) LV mass, (c) Cardiac output, (d) AV peak pressure, and (e) heart/body weight ratio for CD34^{+}Periostin^{+} cell deletion (DT) and non-deletion (No DT) groups (n=6, unpaired two-tailed Student's t-test). Data are presented as the mean ± SEM.

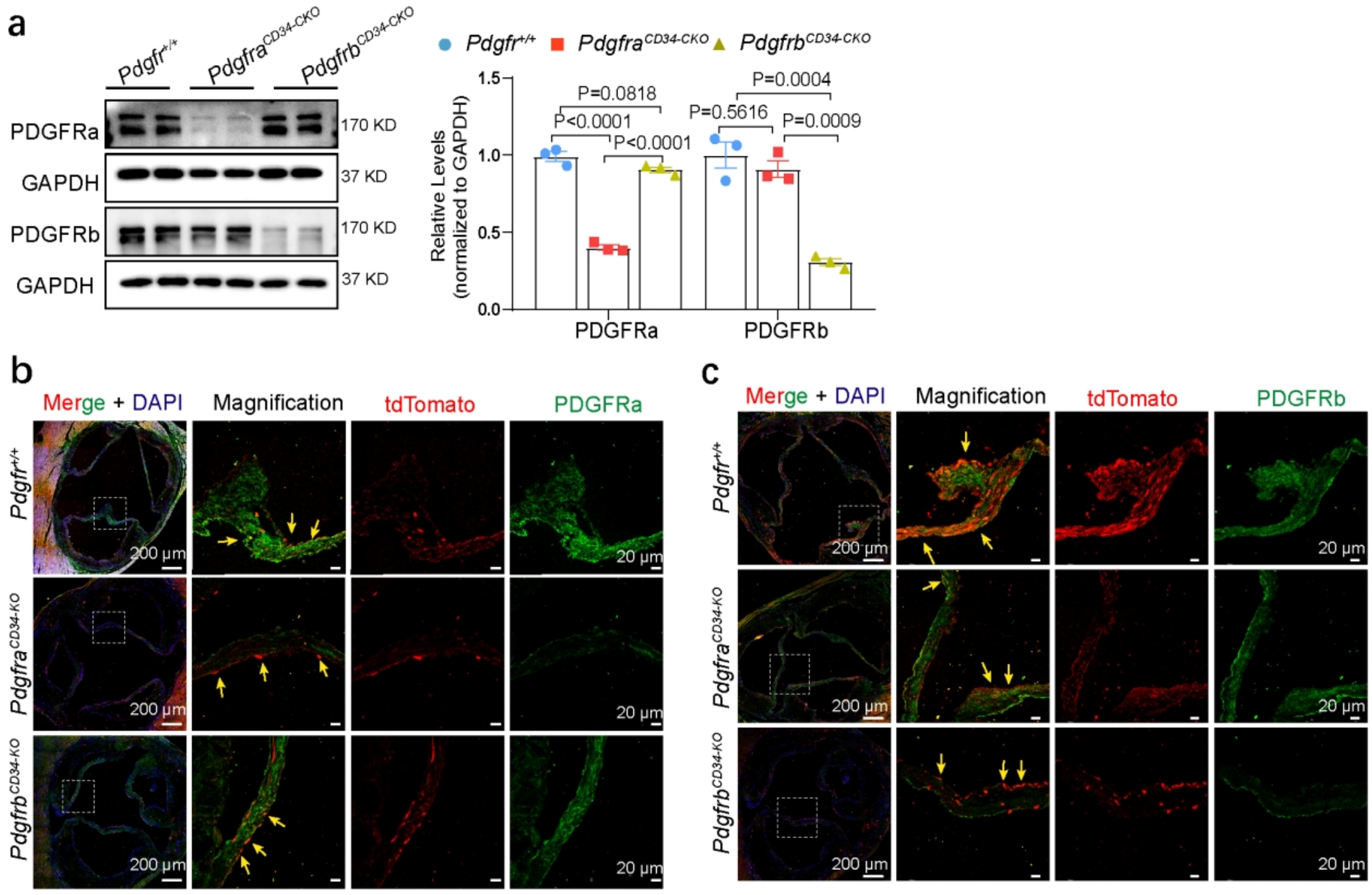

FIGURE S9 (Related to FIGURE 6) Establishment of the CD34^{+} cell specific *Pdgfra/Pdgfrb* knockout lineage tracing mice. (a) Representative western blot of PDGFRa and PDGFRb expression in aortic valve cells (CD34^{+}) isolated from *Pdgfr*$^{+/+}$, *Pdgfra*$^{-/-}$ and *Pdgfrb*$^{-/-}$ mice, respectively. GAPDH served as a loading control (n=3, one-way ANOVA followed by Tukey post hoc test). (b) IF staining of PDGFRa and tdTomato in aortic valvular sections from *Pdgfra*$^{\text{CD34-KO}}$, *Pdgfrb*$^{\text{CD34-KO}}$ and *Pdgfr*$^{+/+}$ mice. Boxed regions are magnified with split channels. Scale bars: 200 μm and 20 μm. (c) IF staining of PDGFRb and tdTomato in aortic valvular sections from *Pdgfra*$^{\text{CD34-KO}}$, *Pdgfrb*$^{\text{CD34-KO}}$ and *Pdgfr*$^{+/+}$ mice. Boxed regions are magnified with split channels. Scale bars: 200 μm and 20 μm.

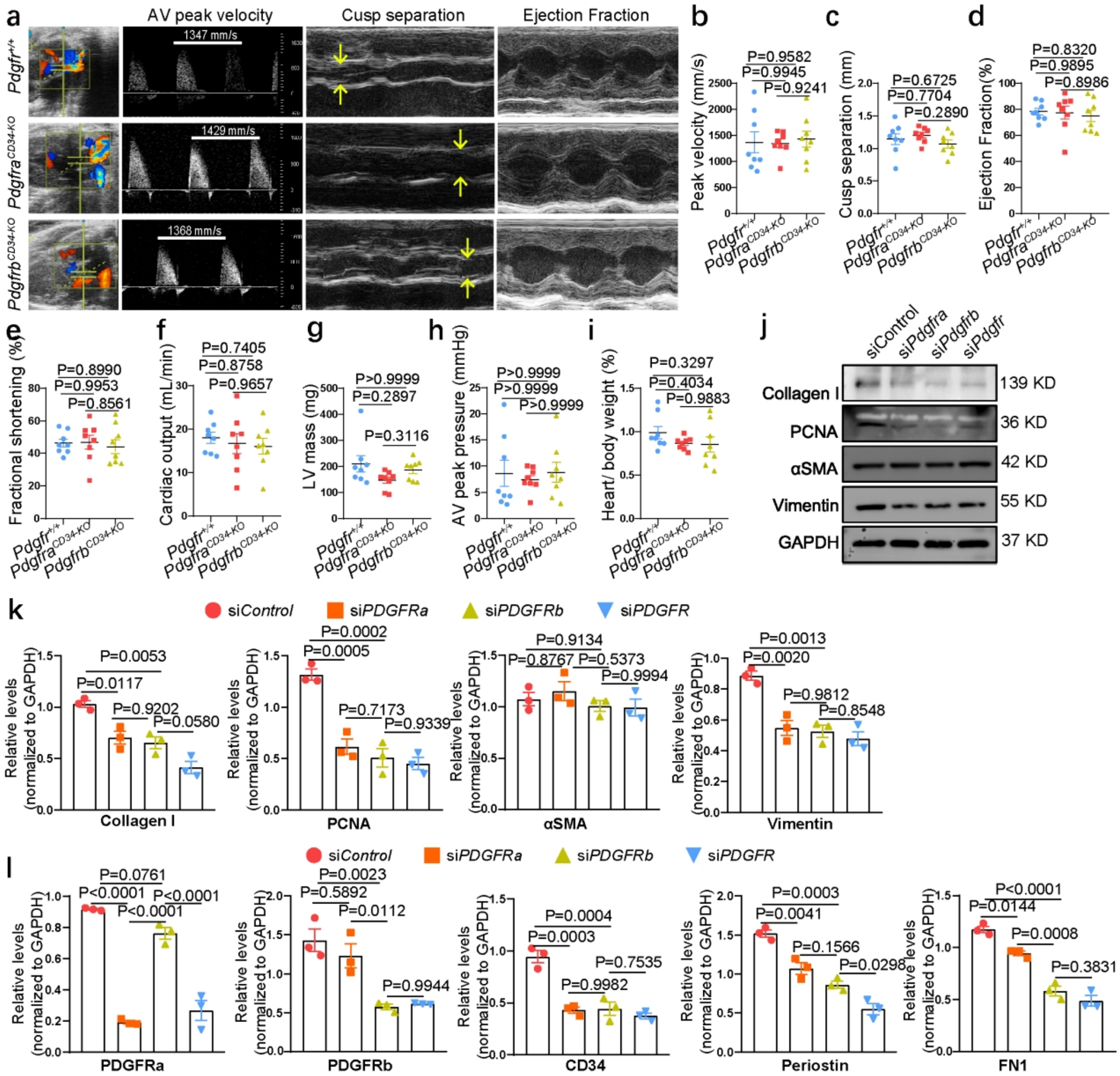

FIGURE S10 (Related to FIGURE 6) Specific deletion of PDGFRa or PDGFRb in $CD34^+$ cells does not alter left ventricular and aortic valve functioning in *Pdgfra*$^{CD34\text{-}KO}$ and *Pdgfrb*$^{CD34\text{-}KO}$ mice. (a) Representative colour Doppler, spectral Doppler, and M-mode in *Cd34*-Dre, *Postn*-CreER^{T2}, and Dou-tdT-DTR tracings obtained from mice with or without DT injection. (b–i) (b) Cusp separation, (c) transaortic peak velocity, (d) ejection fraction, (e) fractional shortening fraction, (f) LV mass, (g) Cardiac output, (h) AV peak pressure, and (i) heart/body weight ratio in indicated groups (n=8, one-way ANOVA followed by Tukey post hoc test). (j) Western blot showing Collagen 1, PCNA, Vimentin, and *α*SMA expression in indicated groups. GAPDH served as a loading control. (k) Quantifications of collagen 1, PCNA, Vimentin, and *α*SMA in indicated groups (n=3, one-way ANOVA followed by Tukey post hoc test). (l) Quantifications of PDGFRa, PDGFRb, CD34, and fibrosis-related proteins in indicated groups (n=3, one-way ANOVA followed by Tukey post hoc test). Related to Figure 6i. Data are presented as the mean ± SEM.

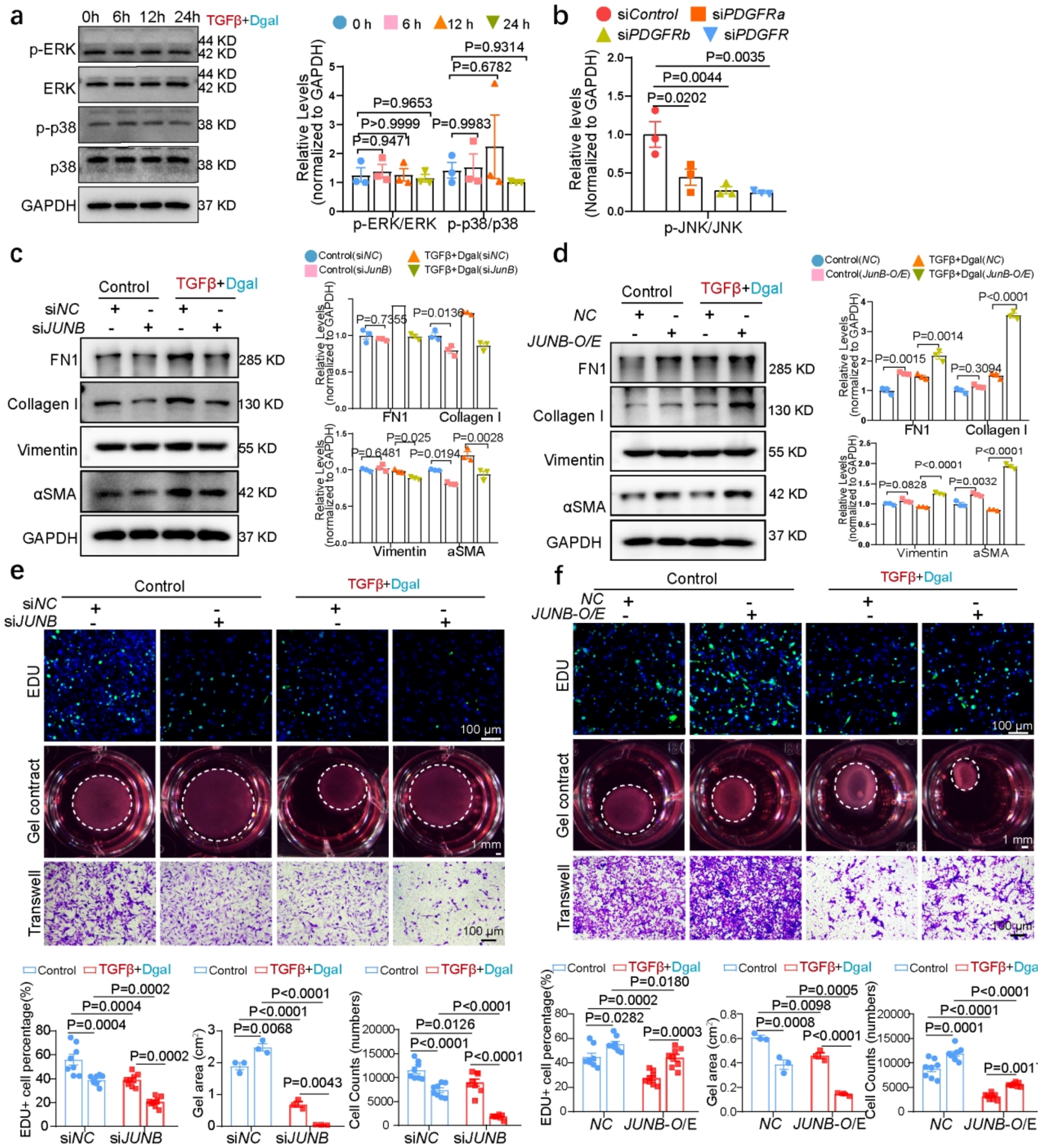

FIGURE S11 (Related to FIGURE 7) Knockdown or overexpression of JUNB regulates proliferation, migration, contractility and the expression of fibrosis-related proteins in CD34⁺ VICs. (a) Western blot detection and quantification of p-ERK, ERK, p-p38, and p38 expression levels in primary CD34⁺ VICs following treatment with TGF-*β* (10 ng/mL) and D-gal (200 μg/mL) for indicated stimulation time (n=3, two-way ANOVA followed by Tukey post hoc test). (b) Quantifications of p-JNK/JNK level in Ctrl, *siPdgfra* and/or *siPdgfrb*-treating primary CD34⁺ VICs, treated with TGF-*β* (10 ng/mL) and D-gal (200 μg/mL) (n = 3, one-way ANOVA followed by Tukey post hoc test); Related to Figure 7d. (c,d) Western blot showing the expression of fibrosis-related proteins in the indicated groups, with GAPDH as a loading control. Quantifications are shown on the right (n = 3, two-way ANOVA followed by Tukey post hoc test). (e,f) EDU assay, collagen gel contraction assay, and transwell assay showing cell

proliferation, migration, and contraction ability respectively. Primary CD34$^+$ VICs cells response to *siJUNB* treatment (e) and *JUNB-O/E* treatment (f), with or without TGF-*β* (10 ng/mL) and D-gal (200 μg/mL) stimulation. Scale bar: 100 μm and 1 mm. Dot-plot located beneath the main panel showing the percentages of EDU$^+$ cell in primary valvular cells (left), quantification (medium) of gel diameters and quantification (right) of cell counts across different groups (n=3-8, two-way ANOVA followed by Tukey post hoc test).

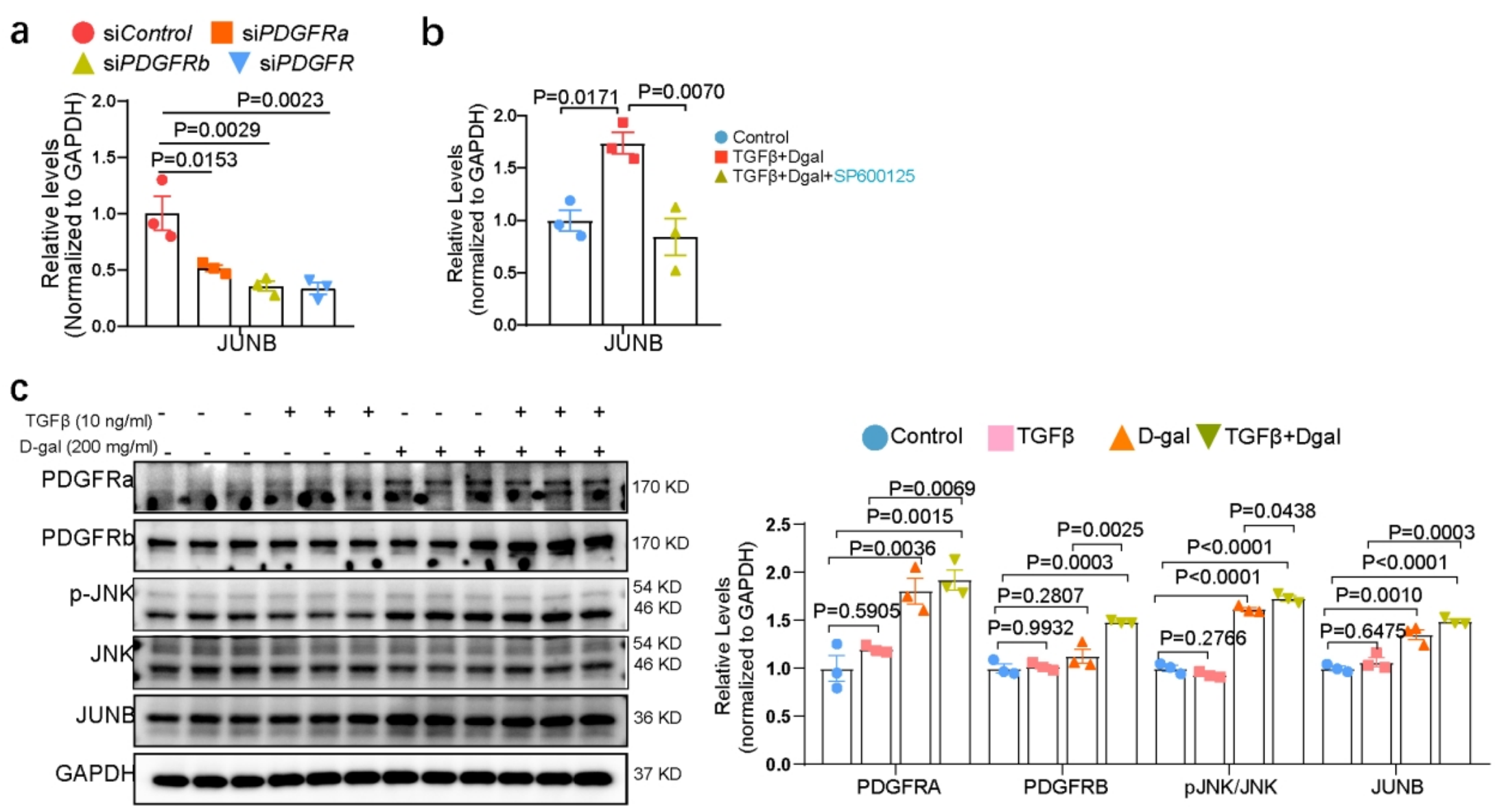

FIGURE S12 (Related to FIGURE 7) D-galactose and TGF-*β* synergistic engagement of the entire PDGFR-JNK-JUNB axis. (a) Quantifications of JUNB level in Ctrl, *siPdgfra* and/or *siPdgfrb*-treating primary CD34$^+$ VICs, treated with TGF-*β* (10 ng/mL) and D-gal (200 μg/mL) (n = 3, one-way ANOVA followed by Tukey post hoc test); Related to Figure 7n. (b) Quantifications of JunB changes in primary CD34$^+$ VICs cells in response to TGF-*β* (10 ng/mL) and D-gal (200 μg/mL) stimulation, with or without Sp600125 (40 μmol/mL) treatment (n = 3, one-way ANOVA followed by Tukey post hoc test); Related to Figure 7o. (c) Western blotting of PDGFRa, PDGFRb, p-JNK, JNK and JUNB in CD34$^+$ VICs, followed by TGF-*β* (10 ng/mL) and/or D-gal (200 μg/mL) stimulation for 12h (n=3, one-way ANOVA followed by Tukey post hoc test). Data are presented as the mean ± SEM.

Materials and Methods

Immunofluorescence staining

Aortic valve samples were embedded in paraffin and sliced into 5 μm thick sections. Paraffin was then removed, samples were rehydrated, and the endogenous peroxidase activity was blocked with 3 % H_2O_2 for 10 min. Antigen

retrieval was performed using 10 mM Tris-EDTA buffer (pH 9.0) at 98 °C for 20 min. Sections were cooled, blocked, and permeabilised using 5 % BSA and 0.1 % Triton X-100 for 1 h, exposed to primary antibodies overnight at 4 °C, and treated with Alexa Fluor-conjugated secondary antibodies (Invitrogen, 1:500) for 1 h at room temperature. Sections were then prepared for staining by washing with PBS, fixing in 4 % PFA for 15 min, permeabilising, and blocking with 5 % bovine serum albumin (BSA) and 0.1 % Triton X-100 for 1 h. Staining was performed as described previously. For *en-face* staining, the aortic valve was carefully dissected from the surrounding tissues and fixed in 4 % PFA and subjected to standard immunostaining before carefully mounting onto slides with the endothelium facing the coverslip. Slides were then compressed with heavy weights overnight at 4 °C to achieve a full stretch, and DAPI was used to counterstain the nuclei, which were mounted in anti-fade medium (Beyotime, P0131). The utilised antibodies are listed in Table S2. Negative controls were constructed using the species-matched IgG isotype controls (Invitrogen, Cat Nos. 31933, 02-6102, 31903, and 31245) for each primary antibody, or by exposure to only the secondary antibody. Fluorescence images of the sections, cultured cells, *en-face* samples, and tissues were captured using a Leica TCS SP8 DIVE confocal microscope.

For tissue clearing and 3D reconstruction, aortic valve tissues were immersed in Cubic-L solution at 37 °C for 2 days, washed with PBS, and incubated with primary antibodies at 4 °C for a further 2 days before treatment with secondary antibodies. The tissues were then cleared in Cubic-R^+ solution and images captured using a Leica confocal microscope. 3D reconstruction was performed with the Imaris software.

Flow cytometric analyses

Bone marrow and blood samples were treated with red blood cell lysis buffer before filtering through a 40-μm cell strainer to obtain a single-cell suspension. Samples were then rinsed with PBS and resuspended in PBS containing 5 % FBS. tdTomato expression was analysed using a BD LSRFortessa II flow cytometer and data were processed using FlowJo v10 software (BD Biosciences).

Data analyses for RNA sequencing

All aortic valve datasets were integrated using Seurat v4.0.1. Integration anchors were identified based on highly variable genes using FindIntegrationAnchors, followed by batch effect correction via canonical correlation analysis (CCA) with IntegrateData. The 'ScaleData' function was used to log-normalise and scale the top 2000 highly variable genes for clustering, which were then subjected to principal component analysis (PCA).

The first 30 principal components were then used to cluster cells and the results visualised using UMAP at a resolution of 0.5.

The clusterProfiler R package (v4.1.7.003) was used for GO enrichment analysis of the DEGS. Terms related to biological processes were analysed and the average expression levels were obtained from the Gene Ontology database (Geneontology. org).

Hisat2 (v2.0.5) and Samtools (v2.10.9) were used to transform FASTQ files into BAM files for bulk RNA sequencing. RNA count expression matrices were obtained using the R subread package (v2.12.3) and DEGs identified using DESeq2 (v1.38.3) based on a count matrix. The count data were then normalised to transcripts per million (TPM) using EdgeR (v3.40.2) for PCA, and plots generated using the “fviz_pca_ind” function in the FactoMineR package (v2.8).

Pseudotime trajectory analyses

The Monocle R package (version 2.14) was used to conduct the pseudo-time trajectory analysis, with default parameters applied unless noted otherwise. To order pseudotime, genes were selected from the top 250 DEGS identified by the differentialGeneTest function and ranked using the average $\log_2$ fold change and adjusted P-value, with the fullModelFormulaStr parameter set to pseudotime. The dimensionality was reduced and the cells aligned with the pseudotime trajectory using the DDRTree method.

EdU in proliferation assay

Primary $CD34^+$ or $CD34^-$ cells were cultured in DMEM containing 10 % FBS and 1 % P/S, with or without the addition of 10 ng/mL TGF-*β* (Novoprotein, CK33) and 200 µg/mL D-gal (Sigma, G0750) for 24 h. EdU (10 µM) was subsequently added and the results incubated for an additional 6 h according to the protocol described in the BeyoClick™ EdU-488 Cell Proliferation Kit (Beyotime, C0071S).

Gel contraction assay

Rat tail collagen I (Good Laboratory Practice Bioscience, GC19587, 200 µL of 5 mg/mL) was mixed with 12 µL of 0.1 mol/L NaOH and 23 µL of 10× PBS. The mixture was then combined with 760 µL of a suspension of primary $CD34^+$ or $CD34^-$ valvular interstitial cells and transferred to culture dishes. After polymerisation at room temperature over 20 min, culture medium with or without TGF-*β* and D-gal was then added, and the dishes incubated at 37 °C. Following 48 h incubation, the gel contraction was evaluated via detachment from the dish walls.

Transwell migration assay

The transwell migration assay was performed using 8.0 μm pore membrane inserts (Corning Costar, 3464) and primary $CD34^+$ or $CD34^-$ valvular interstitial cells (1×10^5 cells per 100 μL serum-free medium) seeded into the upper chambers with or without TGF-*β* and D-gal stimulation. The lower chambers were then filled with 600 μL of DMEM containing 10 % FBS, left to incubate for 24 h, and fixed with 4 % paraformaldehyde before staining with 1 % crystal violet (Beyotime, C0121). Images were captured using a microscope (Olympus, CK53), and the number of migrated cells recorded in five randomly selected fields per well.

Senescence-Associated β-Galactosidase Staining

Primary $CD34^+$ cells were cultured in DMEM with 10% FBS and 1% P/S and treated with or without 10 ng/mL TGF-*β* and 200 μg/mL D-galactose for 48 h. After two PBS rinses, cells were fixed in 4% formaldehyde for 15 min at room temperature, washed three times with PBS (5 min each), and then incubated with freshly prepared staining solution for 36 h using the Senescence-Associated *β*-Gal Staining Kit (Beyotime, C0602) according to the manufacturer's protocol.

Quantitative real-time PCR (qPCR)

TRIzol reagent (Thermo Fisher Scientific, 15596018) was used to isolate total RNA from the cultured cells. Reverse transcription was performed using a RevertAid RT Reverse Transcription Kit (Thermo Fisher Scientific, K1691) to synthesise cDNA. qPCR was performed using the TB Green System (RR820A, TaKaRa Bio). The primer sequences used for qPCR are listed in **Table S3**.

Western Blot

Protein samples of equal volume and concentration were subjected to SDS-PAGE, transferred onto PVDF membranes, and processed according to standard Western blotting protocols. The primary antibodies used are listed in **Table S2**. Pierce ECL western blotting substrates were used to detect chemiluminescent signals, which were captured using a chemiluminescence imaging system (Servicebio) and analysed using Quantity One software. Relative protein levels were standardised using GAPDH or *β*-Actin.
