# Supplementary material for: CD34+ Progenitors Promote Ageing‐Associated Valve Sclerosis through the PDGFR–JNK–JUNB Pathway
Source: Adv Sci (Weinh). 2026 Nov 10:e78607. Online ahead of print. doi: 10.1002/advs.78607 (PMC13752233; doi:10.1002/advs.78607)
Supplement: Supplementary file 3 [file ADVS-9999-e78607-s003.pdf]

Table S1. Baseline Characteristics of Patients

NO.	Age(Years)	Gender (Male/FeM)	Group	Primary diagnosis	BMI（kg/m2）	Patients history	SBP (mmHg)	DBP (mmHg)	Heart rate (beats per	Diabetes mellitus (True/False)	Fasting plasma glucose (mM/L)	Current smoker (True/False)	Former smoker (True/False)	Hyperlipidaemia (True/F	Total cholesterol (mM/L)	Triglycerides (mM/L)	HDL cholesterol (mM/L)	LDL cholesterol (mM/L)	Statins (True/False)	β-Blockers (True/F	ACEi/ARB (True/False)	Bicuspid aortic valves (True/F	Transvalvular mean gradient	Ejection fraction (%)
**1**	30	Male	Non-sclerotic and non-calcific aortic vave Control	Aortic Dissection/ Aortic	24.16	/	100	57	80	F	7.05	F	F	F	3.63	2.01	0.66	2.38	F	T	F	F	9	68
**2**	21	Male	Non-sclerotic and non-calcific aortic vave Control	Marfan syndrome/ Aortic	26.04	/	115	69	79	F	4.88	F	F	F	4.34	1.96	0.98	2.68	F	T	F	F	22	/
**3**	44	Male	Sclerotic aortic valve-sclerosis	Aortic valve regurgitatio	20.18	/	107	45	110	F	5.24	T	T	F	4.35	1.47	0.45	3.23	F	T	F	F	8	/
**4**	55	Male	Sclerotic aortic valve-sclerosis	Aortic Dissection/ Aortic	/	/	137	91	89	F	6.9	F	F	F	3.32	2.85	0.60	2.50	F	T	T	F	4	64
**5**	51	Male	Sclerotic aortic valve-sclerosis	Aortic valve regurgitatio	20.08	/	114	55	81	F	5.07	F	F	F	5.15	0.88	1.58	3.17	F	T	F	F	5	38
**6**	44	Male	Sclerotic aortic valve-sclerosis	Aortic valve regurgitatio	24.21	/	105	54	93	F	3.93	F	T	F	5.16	1.4	0.90	3.71	F	F	F	F	11	65
**7**	80	FeM	Calcific aortic vave-stenosis	Aortic valve stenosis	22.94	/	138	68	87	F	4.78	F	F	F	4.43	4.24	0.97	1.93	F	T	F	F	35	/
**8**	60	Male	Calcific aortic vave-stenosis	Aortic valve stenosis	26.08	Hypertension	115	65	87	F	5.43	T	T	F	3.72	1.39	1.10	2.50	F	T	F	T	52	/
**9**	77	Male	Calcific aortic vave-stenosis	Aortic valve stenosis	24.1	Hypertension	180	56	66	F	5.75	F	F	F	3.13	0.69	1.09	1.86	F	T	T	F	29	66
**10**	75	FeM	Calcific aortic vave-stenosis	Aortic valve stenosis	28.25	Cerebral infarction;Hypert	175	66	72	F	4.36	F	F	F	3.48	2.06	0.91	1.90	F	T	F	F	34	68

SBP, systolic blood pressure; DBP, diastolic blood pressure; HDL, highdensity lipoprotein cholesterol; LDL, low-density lipoprotein cholesterol; NA, not applicable.
Hypertension was defined as a systolic blood pressure over 140 mmHg, and/or a diastolic blood pressure over 90 mmHg. Patients had specific prescription records of anti-hypertensive drugs or currently taking anti-hypertensive medications was also defined as hypertension.
Diabetes was defined as a fasting glucose value over 7.1 mM/L or a history of diabetes medication use, or self-reported in survey of the history of present illness (HPI).
Prior stroke was defined as cerebrovascular accidents caused by focal cerebral, spinal, or retinal infarction.
Hyperlipidemia was defined as elevated blood lipid or lipoprotein (total cholesterol over 6.2mM/L, triglycerides over 2.3mM/L, and/or LDL cholesterol over 4.1mM/L); patients currently taking lipid-lowering medications or previously/ currently prescribed with lipid-lowering medications are also considered to have hyperlipidemia. Medications was self-reported by patients or prescribed by physicians.
