# Supplementary material for: CD34+ Progenitors Promote Ageing‐Associated Valve Sclerosis through the PDGFR–JNK–JUNB Pathway
Source: Adv Sci (Weinh). 2026 Nov 10:e78607. Online ahead of print. doi: 10.1002/advs.78607 (PMC13752233; doi:10.1002/advs.78607)
Supplement: Supplementary file 4 [file ADVS-9999-e78607-s004.pdf]

Table S3. Primer Sequences

Gene	Sequence (5'-3')	Comments
Cd34-CreER^{T2}	GTAGGATAGCAGATGGTCTGGACT<br>CTGTTAATACTCACGCAGCAGACT<br>TGTGCCTCAAATCTATTATTTTGC	Genotyping
R26-tdTomato	CTCTGCTGCCTCCTGGCTTCT<br>CGAGGCGGATCACAAGCAATA<br>TCAATGGGCGGGGGTCGTT	Genotyping
R26-DTR	GCGAAGAGTTTGTCCTCAACC<br>AAAGTCGCTCTGAGTTGTTAT<br>GAGCGGGAGAAATGGATATG	Genotyping
Postn-CreER^{T2}	AGGCATTCAGTCTTTCTTACCCT<br>TCAGTTCCTACCCCACAGGA<br>AGGCACACAAGACTACTTCAA<br>TGCGAACCTCATCACTCGTT	Genotyping
Cd34-Dre	CAACAGGGAGGGGATAAGCC<br>AGCCTCAACTTTGAGCTTTGC<br>AGAGGCAGCCAAGATGACAC<br>AGAGATAGGGAACCAGGCCA	Genotyping
Dou-tdT-DTR	TCAGATTCTTTTATAGGGGACACA<br>TAAAGGCCACTCAATGCTCACTAA<br>ATGAAGCTGCTGCCGTCGG<br>TCAGTGGGAATTAGTCATGCCCAA	Genotyping
Pdgfra-Flox	CAGACATTACTCCAAAGTTAGGCACC<br>TCTTTCCTCACTGCCACCCTCT	Genotyping
Pdgfrb-Flox	GTGATGGACACATGCCAGGTACTA<br>GAGGGGCACATTCTTGCCTGTCTG	Genotyping
Apoe$^{-/-}$	GCCTAGCCGAGGGAGAGCCG<br>TGTGACTTGGGAGCTCTGCAGC<br>GCCGCCCCGACTGCATCT	Genotyping
JUNB	GAGCGCTGGAAGAAGGAGAC<br>GCTTGGACTGGATCACGTTC	qPCR
PERIOSTIN	CAGCAACATGCAGAACCAGA<br>TCCTGGTGCTAAGCAGGTCT	qPCR
GAPDH	ATGGGGAAGGTGAAG GTC G<br>GGGTCATTGATGGCAACAATATC	qPCR
si*Pdgfra*-sense<br>si*Pdgfra*-anti-sense	CAGCGAGUUUAAUGUUUAUTT<br>AUAAACAUUAAACUCGCUGTT	siRNA
si*Pdgfrb*-sense<br>si*Pdgfrb*-anti-sense	GAGCAAGGAUGAAUCUAUATT<br>UAUAGAUUCAUCCUUGCUCTT	siRNA
si*JunB*-sense<br>si*JunB*-anti-sense	AGACCAAGAGCGCAUCAAA<br>UUUGAUGCGCUCUUGGUCU	siRNA
